# Phylogenetic Diversity Indices from an Affine and Projective Viewpoint

**DOI:** 10.1007/s11538-024-01332-x

**Published:** 2024-07-09

**Authors:** V. Moulton, A. Spillner, K. Wicke

**Affiliations:** 1 School of Computing Sciences, University of East Anglia, Norwich, UK; 2 Merseburg University of Applied Sciences, Merseburg, Germany; 3 Department of Mathematical Sciences, New Jersey Institute of Technology, Newark, USA

**Keywords:** Phylogenetic tree, Phylogenetic diversity, Shapley value, Fair Proportion index Cluster system, Split system

## Abstract

Phylogenetic diversity indices are commonly used to rank the elements in a collection of species or populations for conservation purposes. The derivation of these indices is typically based on some quantitative description of the evolutionary history of the species in question, which is often given in terms of a phylogenetic tree. Both rooted and unrooted phylogenetic trees can be employed, and there are close connections between the indices that are derived in these two different ways. In this paper, we introduce more general phylogenetic diversity indices that can be derived from collections of subsets (clusters) and collections of bipartitions (splits) of the given set of species. Such indices could be useful, for example, in case there is some uncertainty in the topology of the tree being used to derive a phylogenetic diversity index. As well as characterizing some of the indices that we introduce in terms of their special properties, we provide a link between cluster-based and split-based phylogenetic diversity indices that uses a discrete analogue of the classical link between affine and projective geometry. This provides a unified framework for many of the various phylogenetic diversity indices used in the literature based on rooted and unrooted phylogenetic trees, generalizations and new proofs for previous results concerning tree-based indices, and a way to define some new phylogenetic diversity indices that naturally arise as affine or projective variants of each other or as generalizations of tree-based indices.

## Introduction

Evolutionary isolation metrics or phylogenetic diversity indices provide quantitative measures of biodiversity and are increasingly popular tools to prioritize species for conservation (Isaac et al. 2007; Redding et al. 2008, 2014; Redding and Mooers 2006; Tucker et al. 2016; Vane-Wright et al. 1991). These indices quantify the importance of a species to overall biodiversity by assessing its unique and shared evolutionary history as indicated by its placement in an underlying phylogeny. Preserving phylogenetic diversity and the “Tree of Life” has become an integral component of conservation considerations (see, e.g., the “Phylogenetic Diversity Task Force”^1^ initiated by the IUCN). Indeed, conservation initiatives like the EDGE of Existence programme^2^ (Gumbs et al. 2023; Isaac et al. 2007) incorporate phylogenetic diversity indices in their identification of species that are both evolutionary distinct and globally endangered. Moreover, the “guide to phylogenetic metrics for conservation, community ecology and macroecology” by Tucker et al. (2016) has been cited more than 700 times since its publication, thus demonstrating an even more widespread interest and application of phylogenetic tools, and in particular different phylogenetic diversity indices, within conservation settings.

Mathematically, with a multitude of phylogenetic diversity indices at hand, there is now an increasing interest in understanding how the different indices relate to each other. Much of the previous work in this direction has focused on comparing and analyzing different indices derived from rooted phylogenetic trees (Bordewich and Semple 2024; Manson 2024; Manson and Steel 2023; Wicke and Steel 2020). Phylogenetic diversity indices have also been defined for unrooted trees (Haake et al. 2008; Wicke and Steel 2020), and an exploration of the relationship between indices derived via rooted and unrooted phylogenetic trees is presented by Wicke and Steel (2020).

**Fig. 1:**
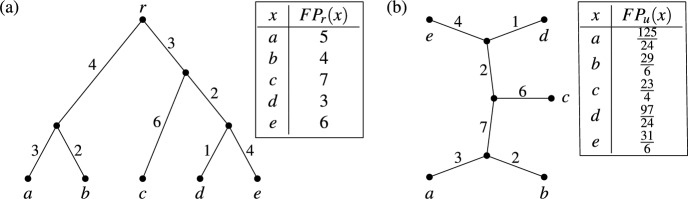
**a** A rooted phylogenetic tree on the set $X = \{a,b,c,d,e\}$ of species. The root vertex is $r$ and all edges are weighted. The table gives the value $FP_r(x)$ of the fair proportion index on this rooted tree for each $x \in X$. **b** The unrooted phylogenetic tree with weighted edges on the same set $X$ of species obtained by suppressing the root of the tree in (**a**). The table gives the value $FP_u(x)$ of the fair proportion index on this unrooted tree for each $x \in X$

As one might expect, phylogenetic diversity indices for rooted and unrooted trees are closely related. To illustrate this, consider the much studied *fair proportion index* (Isaac et al. 2007; Redding 2003). For the rooted phylogenetic tree with edge weights in Fig. 1a, the value $FP_r(x)$ of the rooted fair proportion index for a species $x \in X$ (here and throughout this manuscript, *X* denotes a non-empty finite set of taxa or species) is computed by adding, over all edges that are contained in the path from the root $r$ to the leaf labeled by $x$, the weight of the edge divided by the total number of species for which the path from the root to the leaf labeled by that species also contains that edge. For example, for species $e$ there are three edges in the path from $r$ to $e$ and we obtain

1 $$\begin{aligned} FP_r(e) = \frac{3}{3} + \frac{2}{2} + \frac{4}{1} = 6. \end{aligned}$$

In Wicke and Steel (2020) the fair proportion index has also been defined for unrooted phylogenetic trees. Consider the unrooted phylogenetic tree with edge weights in Fig. 1b. The removal of an edge breaks the tree into two subtrees. The value $FP_u(x)$ of the unrooted fair proportion index for a species $x \in X$ is one half of the value obtained by adding, over all edges in the unrooted tree, the weight of the edge divided by the number of species that lie in the same subtree as $x$ after removal of the edge. For example, for species $e$ we obtain

2 $$\begin{aligned} FP_u(e) = \frac{1}{2} \cdot \left( \frac{3}{4} + \frac{2}{4} + \frac{7}{3} + \frac{6}{4} + \frac{2}{2} + \frac{1}{4} + \frac{4}{1} \right) = \frac{31}{6}. \end{aligned}$$

As can be seen in Fig. 1, $\sum _{x \in X} FP_r(x) = \sum _{x \in X} FP_u(x) = 25$, which is the total weight of the edges of the phylogenetic tree from which the values are computed. Among other natural requirements, this property called *completeness* (formally defined in Sect. 2), should be preserved when relating phylogenetic diversity indices for rooted and unrooted trees.

To better understand how this can be systematically achieved, in this paper we consider indices from the viewpoint of affine and projective clustering. This way of thinking about clustering has its origins in Dress (1997), and since then has become a useful tool in phylogenetic combinatorics (see, e.g., Dress 2012, Ch. 9 and Kleinman et al. 2013). More specifically, in this paper we extend the study of phylogenetic diversity indices into the more general setting of collections of *clusters* (subsets of a set) and collections of *splits* (bipartitions of a set). These settings correspond to affine and projective viewpoints of clustering, respectively (see Sect. 5). Considering collections of clusters and splits in general can be beneficial since it allows for the representation of data that is not tree-like or where it is difficult to determine the correct topology for a phylogenetic tree. Indeed, phylogenetic diversity indices have already been introduced for collections of splits (see, e.g., Abhari et al. 2024).

**Fig. 2:**
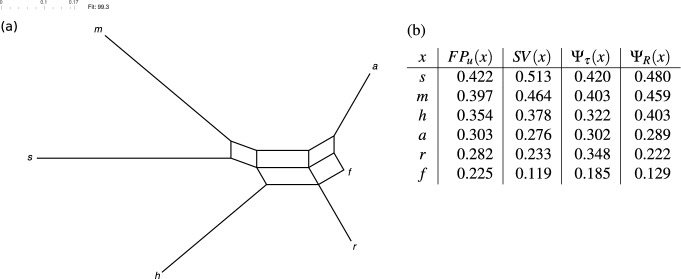
**a** A network visualizing a collection $\mathscr {S}$ of weighted splits on the set $X = \{a,f,h,m,r,s\}$ of six owl populations (see Fig. 12 in the Appendix for more details on this data set). Each band of parallel edges in this network corresponds to a split of $X$ and the length of the edges in the band corresponds to the weight of the split. **b** The values of four different phylogenetic diversity indices $FP_u$, *SV*, $\Psi _{\tau }$ and $\Psi _{R}$ considered in this paper, computed for the six owl populations. The populations are ranked by the values of $FP_u$

To illustrate this way of thinking, as hinted above, collections of clusters naturally arise when computing the rooted fair proportion index. In particular, clusters arise from rooted phylogenetic trees by taking, for each edge, the subset of species for which the path from the root to that species contains the edge (e.g., in Fig. 1a the edge with weight 3 next to the root gives rise to the cluster $\{c,d,e\}$). Thus, the sum used to compute the fair proportion index of *e* in Eq. (1) is just the sum of the values $\frac{\omega (C)}{|C|}$ taken over all clusters *C* that contain *e*, where $\omega (C)$ is the weight of the edge giving rise to cluster *C* and $|C|$ denotes the number of species in $C$. Similarly, we can interpret Eq. (2) in terms of splits, using the fact that splits arise from unrooted phylogenetic trees by taking, for each edge, the split obtained by removing the edge and considering the subsets of species in the two resulting subtrees (e.g., in Fig. 1b the edge with weight 7 gives rise to the split $\{\{a,b\},\{c,d,e\}\}$). Then the sum used to compute the unrooted fair proportion index of *e* in Eq. (2) is just the sum of the values $\frac{\lambda (S)}{2|A|}$ taken over all splits $S$ coming from the tree, where $\lambda (S)$ is the weight of the edge giving rise to $S$ and $A$ is the part in *S* that contains *e*. More generally, the sums used to compute $FP_r$ and $FP_u$ can be applied to *any* collection of weighted clusters or splits, respectively (for example, the values for $FP_u$ computed for a collection of weighted splits visualized by the network in Fig. 2a are shown in the second column in Fig. 2b).

Thinking about phylogenetic diversity indices in an affine and projective way, leads us to two key questions that we will consider in this paper:

(i) How do properties of tree-based phylogenetic diversity indices extend to indices defined via collections of clusters and splits?
(ii) How can the relationships between collections of clusters and collections of splits be exploited to relate cluster- and split-based phylogenetic diversity indices?

In this contribution, we give answers to both of these questions, introducing the concept of phylogenetic diversity indices based on collections of clusters and splits, and giving characterizations for some of these indices in terms of their special properties. We also present a general framework to systematically relate cluster- and split-based phylogenetic diversity indices via a process that is commonly used in phylogenetic combinatorics. This provides concise proofs for generalizations of previous results for trees as well as ways to define new indices.

The rest of this paper is structured as follows. We first illustrate our new concepts and results by focusing on a few well-known tree-based phylogenetic diversity indices, namely the fair proportion index, the Shapely value (Haake et al. 2008; Shapley 1953), and the equal splits index (Redding and Mooers 2006), before we look into some new split-based phylogenetic diversity indices. More specifically, in Sect. 2 we formally define cluster-based phylogenetic diversity indices and present some key properties that such indices may have. Then, in Sect. 3, we present a characterization of the general cluster-based fair proportion index. In Sect. 4 we consider the Shapley value, *SV* (the values of *SV* for the six owl populations considered in Fig. 2a are given in Fig. 2b). In particular, we present a characterization of the Shapley value and use its relationship to the fair proportion index to describe the first building block of our framework. In Sect. 5 we then give the complete framework, and illustrate some of its applications in Sect. 6 using the fair proportion index and a split-based phylogenetic diversity index, $\Psi _{\tau }$, related to the equal splits index as examples. Then, in Sect. 7, we introduce a family of new split-based phylogenetic diversity indices, $\Psi _R$, that generalize the phylogenetic diversity index for unrooted phylogenetic trees given by Wicke and Steel (2020, Sec. 5.2) (the values of the indices $\Psi _{\tau }$ and $\Psi _R$ for the six owl populations considered in Fig. 2a are also given in Fig. 2b). We conclude in Sect. 8 discussing some potential interesting directions for future work.

## Cluster-Based Indices

Let $X$ be a non-empty finite set. We denote the power set of $X$ by $\mathscr {P}(X)$. We call a non-empty subset $C \subseteq X$ a *cluster* on $X$ and call a non-empty collection $\mathscr {C} \subseteq \mathscr {P}(X) {\setminus } \{\emptyset \}$ a *cluster system* on $X$. In this section we introduce the concept of a phylogenetic diversity index on a cluster system, and illustrate some basic properties of these indices by considering a generalization of the fair proportion index for rooted trees that we discussed in the introduction.

To motivate the definition of a phylogenetic diversity index on a cluster system, we briefly look again at rooted phylogenetic trees. Fixing a rooted phylogenetic tree $\mathscr {T}$ on a set $X$ of species, a phylogenetic diversity index $\Phi$ on $\mathscr {T}$ assigns, to each weighting^3^$\omega$ of the edges in $\mathscr {T}$, a vector $\Phi (\omega ) \in \mathbb {R}^X$. To give an example, let $\Phi$ be the fair proportion index on the rooted phylogenetic tree in Fig. 1a. Then, for the weighting $\omega$ of its edges given in Fig. 1a, we can write

3 $$\begin{aligned} \Phi (\omega ) = (5,4,7,3,6), \end{aligned}$$

or, in more detail, $(\Phi (\omega ))(a) = 5, \ (\Phi (\omega ))(b) = 4, \ldots ,\ (\Phi (\omega ))(e) = 6$.

**Fig. 3:**
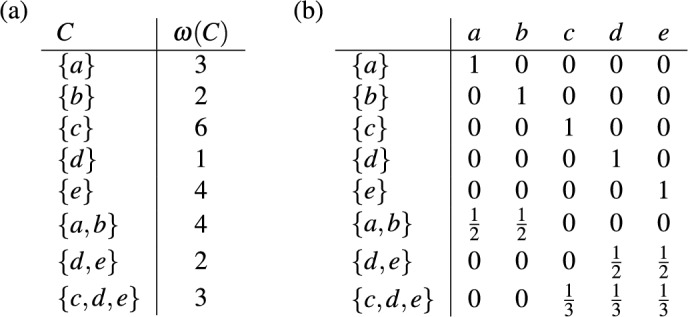
**a** The weighted clusters on $X$ corresponding to the edges of the rooted phylogenetic tree in Fig. 1a. **b** The matrix $\Gamma$ from Eq. (5) for the fair proportion index on $\mathscr {C}$, where $\mathscr {C}$ is the cluster system consisting of the clusters given in (**a**)

As described in the introduction, each edge in a rooted phylogenetic tree on $X$ is associated with a cluster on *X*. In Fig. 3a the clusters associated with the edges of the rooted phylogenetic tree in Fig. 1a are given, where each cluster is weighted by the length of the corresponding edge. Note that this cluster system $\mathscr {C}$ has a special property, namely it is a *hierarchy*, that is, $C \cap C' \in \{\emptyset ,C,C'\}$ holds for all $C,C' \in \mathscr {C}$. In particular, as we see in this example, hierarchies are essentially those cluster systems that can be represented by a rooted phylogenetic tree on $X$ [see, e.g., Semple and Steel (2003, Thm. 3.5.2) for a more precise statement of this fact using the concept of a rooted $X$-tree].

Bearing these facts in mind, for an arbitrary cluster system $\mathscr {C}$ on $X$, we consider the space $\mathbb {L}(\mathscr {C})$ consisting of all weightings $\omega : \mathscr {C} \rightarrow \mathbb {R}$. We then define a *phylogenetic diversity index* on $\mathscr {C}$ to be a map $\Phi : \mathbb {L}(\mathscr {C}) \rightarrow \mathbb {R}^X$. For example, following the intuitive description in the introduction, we define the *fair-proportion index* on a cluster system $\mathscr {C}$ on *X* by putting, for each $\omega \in \mathbb {L}(\mathscr {C})$ and all $x \in X$,

4 $$\begin{aligned} (FP(\omega ))(x)&= \sum \limits _{C \in \mathscr {C}: \ x \in C} \frac{\omega (C)}{\vert C \vert }. \end{aligned}$$

It can then be checked that (4) applied to the weighted cluster system in Fig. 3a yields precisely the vector we saw in (3).

We now introduce three key properties of cluster-based indices which generalize properties of tree-based indices described in the literature. We will illustrate these properties for the fair proportion index and, as we shall see, these properties are also shared by some of the other phylogenetic diversity indices that we consider later on.

Let $\mathscr {C}$ be a cluster system on $X$. A phylogenetic diversity index $\Phi$ on $\mathscr {C}$ is *additive* if

(A) $$\Phi (\omega _1 + \omega _2) = \Phi (\omega _1) + \Phi (\omega _2)$$ for all $$\omega _1, \omega _2 \in \mathbb {L}(\mathscr {C})$$,

and $\Phi$ is *homogeneous* if

(H) $\Phi (a \cdot \omega ) = a \cdot \Phi (\omega )$ for all $\omega \in \mathbb {L}(\mathscr {C})$ and all $a \in \mathbb {R}$.

Properties (A) and (H) together mean that $\Phi$ is a *linear map*, in which case we call $\Phi$ *linear*. Phylogenetic diversity indices considered in the literature are usually linear. This may be due to useful consequences of linearity such as, for example, that applying a linear phylogenetic diversity index to a weighting obtained by taking the average over several different edge weightings of a fixed rooted phylogenetic tree amounts to averaging the values of the phylogenetic diversity index. In this paper, most (but not all) results assume linearity of the phylogenetic diversity indices involved. To avoid any confusion, we will always explicitly state which properties we assume.

Note that every linear phylogenetic diversity index $\Phi$ on $\mathscr {C}$ corresponds to a $|\mathscr {C}| \times |X|$-matrix $\Gamma = \Gamma _{\Phi } = (\gamma _{(C,x)})$ such that

5 $$\begin{aligned} (\Phi (\omega ))(x) = \sum _{C \in \mathscr {C}} \omega (C) \cdot \gamma _{(C,x)} \end{aligned}$$

for all $\omega \in \mathbb {L}(\mathscr {C})$ and all $x \in X$. The entries of the matrix $\Gamma$ are usually assumed to be non-negative (see, e.g., Manson and Steel 2023, Def. 1). Again, our framework also applies when this assumption is violated. In Sect. 7 we will come back to this point. In Fig. 3b we give, as an example, the matrix $\Gamma$ corresponding to the fair proportion index on the cluster system in Fig. 3a.

Finally, we call a phylogenetic diversity index $\Phi$ on $\mathscr {C}$ *complete* if

(C) $\sum _{x \in X} (\Phi (\omega ))(x) = \sum _{C \in \mathscr {C}} \omega (C)$ holds for all $\omega \in \mathbb {L}(\mathscr {C})$.

For tree-based phylogenetic diversity indices, completeness is often required as part of their definition (see, e.g., Bordewich and Semple 2024; Wicke and Steel 2020). For example, we have seen in the introduction for the fair proportion index on a rooted phylogenetic tree that $\sum _{x \in X} FP_r(x)$ equals the total weight of the edges in the tree. Property (C) expresses this fact in terms of clusters. Note that a linear phylogenetic diversity index $\Phi$ on $\mathscr {C}$ is complete if and only if $\sum _{x \in X} \gamma _{(C,x)} = 1$ for all $C \in \mathscr {C}$ (cf. Wicke and Steel 2020, Eq. (2) and Wicke 2020, Eq. (1)), where $\Gamma = (\gamma _{(C,x)})$ is the matrix from Eq. (5).

We next show that the fair proportion index satisfies all three of the above properties.

### Lemma 2.1

The fair proportion index is a complete, linear phylogenetic diversity index on $\mathscr {C}$ for any cluster system $\mathscr {C}$ on $X$.

### Proof

As we have seen in the example in Fig. 3, the fair proportion index can be described by a matrix $\Gamma = (\gamma _{(C,x)})$ where the row associated with a cluster $C \in \mathscr {C}$ contains $|C|$ entries equal to $\frac{1}{|C|}$ and $|X| - |C|$ entries equal to 0. $\square$

**Fig. 4:**
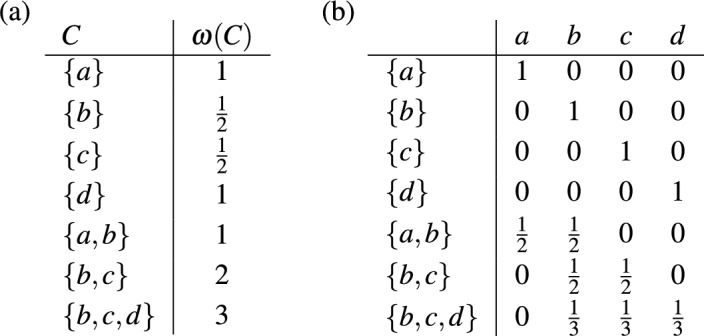
**a** A weighted cluster system ${{\mathscr {C}}}$ on $X=\{a,b,c,d\}$ that is not a hierarchy. **b** The matrix $\Gamma$ from Eq. (5) for the fair proportion index $\Phi$ on $\mathscr {C}$

We conclude this section with an example of a weighted cluster system $\mathscr {C}$ on $X=\{a,b,c,d\}$ that is not a hierarchy and which illustrates the possible consequences of restricting $\mathscr {C}$ to some hierarchy. The cluster system $\mathscr {C}$ and the weighting $\omega$ are given in Fig. 4a. Figure 4b gives the matrix $\Gamma =\Gamma _{\Phi }$ corresponding to the fair proportion index $\Phi$ on $\mathscr {C}$. We have $\Phi (\omega ) = \left( \frac{3}{2}, 3, \frac{5}{2}, 2 \right)$. Now consider the hierarchies ${{\mathscr {C}}}_1 = {{\mathscr {C}}}\setminus \{ \{a,b\} \}$ and ${{\mathscr {C}}}_2 = {{\mathscr {C}}}{\setminus } \{ \{b,c\}, \{b,c,d\} \}$ and the fair proportion index $\Phi _i$ on $\mathscr {C}_i$, $i \in \{1,2\}$. Note that the matrix $\Gamma _{\Phi _i}$ is obtained from $\Gamma$ by removing the rows corresponding to clusters in $\mathscr {C} {\setminus } \mathscr {C}_i$ and the weighting $\omega _i$ is just the restriction of $\omega$ to $\mathscr {C}_i$. This yields $\Phi _1(\omega _1) = \left( \frac{3}{2}, 1, \frac{1}{2}, 1 \right)$ and $\Phi _2(\omega _2) = \left( 1, \frac{5}{2}, \frac{5}{2}, 2 \right)$. As can be seen, the rankings of the elements in $X$ obtained by $\Phi _1$ and $\Phi _2$ are different and, thus, need not coincide with the ranking obtained by considering the whole cluster system $\mathscr {C}$.

## A Characterization of the Fair Proportion Index

In general, it is of interest to characterize phylogenetic diversity indices in terms of their key properties, as this can help to understand better how they are related to one another. In this section, as an illustration for cluster-based indices, we shall present a characterization of the fair proportion index. This generalizes the characterization of the fair proportion index on rooted phylogenetic trees given by Manson and Steel (2023, Thm. 6).

Our characterization will require three properties. The first two properties concern linear phylogenetic diversity indices $\Phi$ on a cluster system $\mathscr {C}$ on $X$, and are given in terms of the matrix corresponding to $\Phi$. For all $C \in \mathscr {C}$, let $ch(C)$ denote the set of those $C' \in \mathscr {C}$ with $C' \subsetneq C$ such that there is no $C'' \in \mathscr {C}$ with $C' \subsetneq C'' \subsetneq C$. We emphasize that even though a cluster in $\mathscr {C}$ may receive the weight 0, it is still considered as present in $\mathscr {C}$ and, therefore, the sets $ch(C)$ for $C \in \mathscr {C}$ do not change when such a weighting is encountered.

We say that $\Phi$ satisfies the *neutrality condition* if

(NC) the entries of the matrix $\Gamma _{\Phi }$ in Eq. (5) are such that $\gamma _{(C,x)} = \gamma _{(C,y)}$ holds for all $C \in \mathscr {C}$ with $ch(C) = \emptyset$ and all $x,y \in C$.

A property similar to (NC) was introduced by Manson and Steel (2023) for rooted $X$-trees. In addition, we say that $\Phi$ is a *descendant diversity index* if

(DD) $\Phi$ is complete, all entries of the matrix $\Gamma _{\Phi }$ in Eq. (5) are non-negative and, for all $C \in \mathscr {C}$, $\gamma _{(C,x)} = 0$ if $x \not \in C$.

Property (DD) was introduced by Bordewich and Semple (2024) for the special case where the cluster system $\mathscr {C}$ is a hierarchy (using the equivalent description of hierarchies in terms of rooted $X$-trees).

**Fig. 5:**
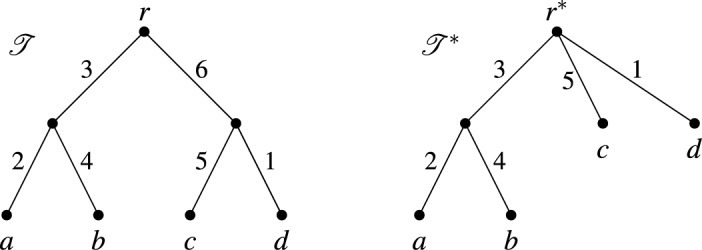
Collapsing the edge with weight $6$ in the rooted phylogenetic tree $\mathscr {T}$ on $X=\{a,b,c,d\}$ yields the rooted phylogenetic tree $\mathscr {T}^*$ on $X$

The third property is a bit more complicated, and thus we first motivate it using rooted trees as in Manson and Steel (2023). Let $\mathscr {T}$ be a rooted phylogenetic tree on $X$ with edge weights and let $\mathscr {T}^*$ be the rooted phylogenetic tree on $X$ obtained by collapsing one of the edges of $\mathscr {T}$. This is illustrated in Fig. 5. In addition, let $\Phi$ and $\Phi ^*$ be phylogenetic diversity indices on $\mathscr {T}$ and $\mathscr {T}^*$, respectively. Both $\Phi$ and $\Phi ^*$ yield a vector in $\mathbb {R}^X$ for all weightings of the edges of $\mathscr {T}$ and $\mathscr {T}^*$, respectively. The topology of the rooted phylogenetic trees, however, may have an impact on how the weights of the edges are used to compute these vectors by $\Phi$ and $\Phi ^*$, respectively. Therefore, since the topologies of $\mathscr {T}$ and $\mathscr {T}^*$ differ, the vector in $\mathbb {R}^X$ that we obtain by $\Phi ^*$ for $\mathscr {T}^*$ will usually not coincide with the vector that we obtain by $\Phi$ in the limit, as the weight of the edge in $\mathscr {T}$ tends to 0 (keeping the weights of all other edges in $\mathscr {T}$ in constant).

With this in mind, let $\mathscr {C}$ be a cluster system on $X$ and let $C \in \mathscr {C}$ be such that $\mathscr {C}^* = \mathscr {C} {\setminus } \{C\}$ is non-empty. A phylogenetic diversity index $\Phi$ on $\mathscr {C}$ is *downward continuous* with respect to a phylogenetic diversity index $\Phi ^*$ on $\mathscr {C}^*$ if

(DC) for all $\omega \in \mathbb {L}(\mathscr {C})$ we have
  6 $$\begin{aligned} \lim _{\omega (C) \rightarrow 0} \Phi (\omega ) = \Phi ^*(\omega ^*), \end{aligned}$$

where $\omega ^* \in \mathbb {L}(\mathscr {C}^*)$ is the weighting with $\omega ^*(D) = \omega (D)$ for all $D \in \mathscr {C}^*$. Note that when forming the cluster system $\mathscr {C}^*$ we remove the cluster *C* from the cluster system $\mathscr {C}$ but not the elements in $C$ from the set $X$. In particular, both $\Phi$ and $\Phi ^*$ yield vectors in $\mathbb {R}^X$.

With the properties (NC), (DD) and (DC) in hand, we now present our characterization of the fair proportion index.

### Theorem 3.1

Suppose we have, for each cluster system $\mathscr {C}$ on $X$, a phylogenetic diversity index $\Phi _{\mathscr {C}}$ on $\mathscr {C}$. Then the following are equivalent:

(i) For all cluster systems $\mathscr {C}$ on $X$, $\Phi _{\mathscr {C}}$ is the fair proportion index on $\mathscr {C}$.
(ii) For all cluster systems $\mathscr {C}$ on $X$, $\Phi _{\mathscr {C}}$ is a descendant diversity index that satisfies the neutrality condition and is downward continuous with respect to $\Phi _{\mathscr {C} {\setminus } \{C\}}$ for all $C \in \mathscr {C}$ such that $\mathscr {C} {\setminus } \{C\} \ne \emptyset$.

Before proving this theorem, to provide some intuition for its statement, consider the cluster system ${{\mathscr {C}}}= \{ \{a,b\}, \{a,b,c,d\}, \{a,b,c,d,e\}\}$ on $X=\{a,b,c,d,e\}$, and let $\Phi$ be the linear phylogenetic diversity index with the following matrix $\Gamma _\Phi$

$$\begin{aligned} \begin{array}{r |ccccc} ~ &{} a &{} b &{} c &{} d &{} e \\ \hline \{a,b\} &{} 1/2 &{} 1/2 &{} 0 &{} 0 &{} 0\\ \{a,b,c,d\} &{} 1/4 &{} 1/4 &{} 1/4 &{} 1/4 &{} 0 \\ \{a,b,c,d,e\} &{} 2/5 &{} 2/5 &{} 1/15 &{} 1/15 &{} 1/15. \\ \end{array} \end{aligned}$$

In addition, let $C=\{a,b\}$, $\mathscr {C}^* = \mathscr {C} {\setminus } \{C\}$, and let $\Phi ^*$ be the linear phylogenetic diversity index on $\mathscr {C}^*$ whose matrix $\Gamma _{\Phi ^*}$ is obtained by deleting the row corresponding to $C$ from $\Gamma _{\Phi }$. Then both $\Phi$ and $\Phi ^*$ satisfy properties (NC) and (DD). Moreover, $\Phi$ is downward continuous with respect to $\Phi ^*$. But, clearly, $\Phi$ is not the fair proportion index on $\mathscr {C}$. Hence, it is not enough to look at a phylogenetic diversity index $\Phi$ on a cluster system $\mathscr {C}$ and phylogenetic diversity indices $\Phi ^*$ on cluster systems $\mathscr {C} {\setminus } \{C\}$ for some $C \in \mathscr {C}$. Instead we need to look at *all* cluster systems on $X$.

### Proof of Theorem 3.1

We first show that (i) implies (ii). Consider a cluster system $\mathscr {C}$ on $X$ and put $\Phi = \Phi _{\mathscr {C}}$. By assumption, $\Phi$ is the fair proportion index on $\mathscr {C}$. Thus, in view of Lemma 2.1, $\Phi$ is linear and complete. Moreover, as illustrated by the example in Fig. 3b, it follows immediately from the definition of the fair proportion index in (4) that $\Phi$ is a descendant diversity index and satisfies the neutrality condition.

It remains to establish downward continuity. Consider a cluster $C \in \mathscr {C}$ and assume that $\mathscr {C}^* = \mathscr {C} {\setminus } \{C\} \ne \emptyset$. Put $\Phi ^* = \Phi _{\mathscr {C}^*}$. Let $\Gamma = \Gamma _{\Phi }$ and $\Gamma ^* = \Gamma _{\Phi ^*}$ be the matrices whose entries satisfy Eq. (5) for $\Phi$ and $\Phi ^*$, respectively. By assumption, $\Phi$ is the fair proportion index on $\mathscr {C}$ and $\Phi ^*$ is the fair proportion index on $\mathscr {C}^*$. Therefore, it follows again from the definition of the fair proportion index in (4) that deleting the row corresponding to the cluster $C$ from the matrix $\Gamma$ yields the matrix $\Gamma ^*$. But this immediately implies that Eq. (6) holds for all $\omega \in \mathbb {L}(\mathscr {C})$, as required.

Next we show that (ii) implies (i). Let $\mathscr {C}$ be a cluster system on $X$. By assumption, $\Phi = \Phi _{\mathscr {C}}$ is a descendant diversity index and, therefore, linear. Let $\Gamma = \Gamma _{\Phi }$ be the matrix whose entries satisfy Eq. (5) for $\Phi$. In view of the definition of the fair proportion index in (4), it suffices to show that the entries of $\Gamma$ satisfy

$$\begin{aligned} \gamma _{(C,x)} = {\left\{ \begin{array}{ll} \frac{1}{|C|} &{}\text {for} \ x \in C\\ 0 &{}\text {for} \ x \not \in C \end{array}\right. } \end{aligned}$$

for all $C \in \mathscr {C}$ and all $x \in X$. We use induction on $|\mathscr {C}|$ to show this.

To establish the base case of the induction, assume $|\mathscr {C}| = 1$. Consider $C \in \mathscr {C}$ and $x \in X$. In view of $|\mathscr {C}| = 1$ we have $ch(C) = \emptyset$. Thus, in view of the assumption that $\Phi$ is a descendant diversity index and satisfies the neutrality condition, we have $\gamma _{(C,x)}=\frac{1}{|C|}$ for all $x \in C$ and $\gamma _{(C,x)}=0$ for all $x \in X {\setminus } C$, as required.

Next assume $|\mathscr {C}| \ge 2$. Consider $C \in \mathscr {C}$ and put $\mathscr {C}^* = \mathscr {C} {\setminus } \{C\}$. By the assumption that $\Phi$ is downward continuous with respect to $\Phi ^* = \Phi _{\mathscr {C}^*}$, the matrix $\Gamma ^* = \Gamma _{\Phi ^*}$ whose entries satisfy Eq. (5) for $\Phi ^*$ is obtained by deleting the row corresponding to cluster $C$ from $\Gamma$. Thus, by induction, we have

$$\begin{aligned} \gamma _{(D,x)} = {\left\{ \begin{array}{ll} \frac{1}{|D|} &{}\text {for} \ x \in D\\ 0 &{}\text {for} \ x \not \in D \end{array}\right. } \end{aligned}$$

for all $D \in \mathscr {C} {\setminus } \{C\}$ and all $x \in X$. Since this holds for all $C \in \mathscr {C}$, this finishes the inductive proof. $\square$

## The Shapely Value

The Shapely value is a well-known phylogenetic diversity index that can be computed using rooted phylogenetic trees and that has its origins in game theory. Interestingly, to understand a generalization of this index in the cluster setting, it is necessary to consider mappings on slightly more general spaces than those used in the definition of cluster-based phylogenetic diversity indices in Sect. 2. In this section, we shall explain this, and then give a characterization of a cluster-based version of the Shapely value.

As before, let $X$ be a finite non-empty set. A *game* is a map $g: \mathscr {P}(X) \rightarrow \mathbb {R}$. The elements of $X$ are referred to as the *players* in this context and the value $g(C)$ for some $C \in \mathscr {P}(X)$ can be interpreted as the gain when the players in $C$ form a coalition. One aspect of analyzing such a game is to quantify, for each player $x \in X$, the value $v(x) \in \mathbb {R}$ of the player with respect to the game (see, e.g., Branzei et al. 2008 for a more detailed exposition of these concepts).

Formally speaking, we are thus interested in maps $v$ from $\mathbb {R}^{\mathscr {P}(X)}$ to $\mathbb {R}^X$, and the *Shapley value* is one specific such map $v$ given by

7 $$\begin{aligned} (v(g))(x) = \frac{1}{|X|!} \cdot \sum _{M \in \mathscr {P}(X): \ x \in M} [(|M|-1)! \cdot (|X|-|M|)! \cdot (g(M)-g(M \setminus \{x\}))].\nonumber \\ \end{aligned}$$

This map was originally proposed by Shapley (1953).

In a biological context, the players of Shapley’s game are species and from a rooted phylogenetic tree $\mathscr {T}$ on $X$ with edge weights we obtain a game *g* by setting $g(M)=PD(M)$ for each $M \in \mathscr {P}(X)$, where *PD*(*M*) is the *phylogenetic diversity* of $M$. The value *PD*(*M*) is defined as the total weight of those edges in $T$ that lie on a path from the root to some species in $M$ (Faith 1992). For example, for the rooted phylogenetic tree in Fig. 1a we obtain

$$\begin{aligned}PD(\{a,b,d\}) = 3+2+4+3+2+1 = 15.\end{aligned}$$

We now explain a way to generalize these considerations to cluster systems $\mathscr {C}$ on $X$. First we need to define the phylogenetic diversity of a subset of *X* relative to a weighted cluster system. Let $\omega \in \mathbb {L}(\mathscr {C})$. Then the *phylogenetic diversity* of a subset $M$ of $X$ with respect to $\omega$ is defined as

8 $$\begin{aligned} PD(M) = PD_{\omega }(M) = \sum _{C \in \mathscr {C}: \ M \cap C \ne \emptyset } \omega (C). \end{aligned}$$

To further explore properties of the Shapley value in the context of our work, it will be convenient to consider the set

$$\begin{aligned}\mathbb{P}\mathbb{D}(\mathscr {C}) = \{g \in \mathbb {R}^{\mathscr {P}(X)}: \ \text {there exists} \ \omega \in \mathbb {L}(\mathscr {C}) \ \text {with} \ g = PD_{\omega }\},\end{aligned}$$

that is, the set of games in $\mathbb {R}^{\mathscr {P}(X)}$ for which there is some $\omega \in \mathbb {L}(\mathscr {C})$ which gives rise to this game.

The following lemma states two key structural properties of the set $\mathbb{P}\mathbb{D}(\mathscr {C})$ for any cluster system $\mathscr {C}$ on $X$. To prove this lemma, we define, for all $C \in \mathscr {P}(X)$, the game $g_C: \mathscr {P}(X) \rightarrow \mathbb {R}$ obtained by putting

9 $$\begin{aligned} g_C(M) = {\left\{ \begin{array}{ll} 1 &{}\text {if} \ C \cap M \ne \emptyset \\ 0 &{}\text {if} \ C \cap M = \emptyset . \end{array}\right. } \end{aligned}$$

### Lemma 4.1

Let $\mathscr {C}$ be a cluster system on $X$. Then $\mathbb{P}\mathbb{D}(\mathscr {C})$ is a linear subspace of $\mathbb {R}^{\mathscr {P}(X)}$ that has dimension $|\mathscr {C}|$.

### Proof

In view of (8), $\mathbb{P}\mathbb{D}(\mathscr {C})$ is the linear span of the games $g_C$ for $C \in \mathscr {C}$ defined in (9):

$$\begin{aligned} PD_{\omega }(M) = \sum _{C \in \mathscr {C}: \ M \cap C \ne \emptyset } \omega (C) = \sum _{C \in \mathscr {C}} \omega (C) \cdot g_{C}(M) \end{aligned}$$

Thus, it suffices to show that the games $g_C$, $C \in \mathscr {C}$, are linearly independent. To see this, consider the square matrix $A$ whose rows and columns are each in one-to-one correspondence with the elements of $\mathscr {P}(X) {\setminus } \{\emptyset \}$. For all $C,M \in \mathscr {P}(X) {\setminus } \{\emptyset \}$ the entry of $A$ in the row corresponding to $C$ and the column corresponding to $M$ is 1 if $C \cap M \ne \emptyset$ and is 0 otherwise. $A$ is the so-called *intersection matrix* of $\mathscr {P}(X) {\setminus } \{\emptyset \}$ and it is known that $A$ has full rank (see, e.g., Jukna 2011, p. 216). Thus, in particular, the rows corresponding to $C \in \mathscr {C}$ are linearly independent. $\square$

Now, as explained above, for a cluster system $\mathscr {C}$ on $X$, we restrict in (7) to games $g=PD$ in $\mathbb{P}\mathbb{D}(\mathscr {C})$. More specifically, we define the Shapley value *relative to the cluster system* $\mathscr {C}$ as the map $SV: \mathbb{P}\mathbb{D}(\mathscr {C}) \rightarrow \mathbb {R}^X$ obtained by putting

10 $$\begin{aligned} (SV(PD))(x){} & {} = \frac{1}{|X|!} \cdot \sum _{M \in \mathscr {P}(X): \ x \in M} \left[ (|M|-1)! \cdot (|X|-|M|)! \cdot (PD(M)\right. \nonumber \\{} & {} \quad \left. -PD(M \setminus \{x\})) \right] \end{aligned}$$

for all $PD \in \mathbb{P}\mathbb{D}(\mathscr {C})$ and all $x \in X$. Note that, in view of Lemma 4.1, $\mathbb{P}\mathbb{D}(\mathscr {C})$ may be a proper subspace of $\mathbb {R}^{\mathscr {P}(X)}$ (i.e., the set of all games). As we will see below, any characterization of the Shapley value relative to a cluster system must take this into account (see also Dubey 1975 for a more general discussion of this aspect).

The sharp-eyed reader will have noticed that the Shapley value relative to a cluster system $\mathscr {C}$ is *not* a phylogenetic diversity index on $\mathscr {C}$, as the latter is defined as a map from $\mathbb {L}(\mathscr {C})$ to $\mathbb {R}^X$. However, we can resolve this issue by slightly generalizing our cluster-based definition of phylogenetic diversity indices. Let $\mathbb {L}$ be a linear subspace of $\mathbb {R}^{\mathscr {P}(X)}$. Then we define a phylogenetic diversity index *on* $\mathbb {L}$ to be a map $\Phi : \mathbb {L} \rightarrow \mathbb {R}^X$. This encompasses then the Shapley value as a phylogenetic diversity index on $\mathbb {L} = \mathbb{P}\mathbb{D}(\mathscr {C})$ for all cluster systems $\mathscr {C}$ on $X$. Moreover, viewing $\mathbb {L}(\mathscr {C})$ as the linear subspace

$$\begin{aligned}\mathbb {L} = \{\omega \in \mathbb {R}^{\mathscr {P}(X)}: \omega (C)=0 \ \text {for all} \ C \not \in \mathscr {C}\},\end{aligned}$$

it also encompasses phylogenetic diversity indices on $\mathscr {C}$ as defined in Sect. 2. In fact, we can say even more about these relationships, which we will return to in the next section.

For the remainder of this section, we focus on giving a characterization of the Shapley value relative to a cluster system. This will involve the following two properties. We say that a phylogenetic diversity index $\Phi$ on a linear subspace $\mathbb {L}$ of $\mathbb {R}^{\mathscr {P}(X)}$ satisfies *Pareto efficiency* if

(PE) $\sum _{x \in X} (\Phi (\omega ))(x) = \omega (X)$ for all $\omega \in \mathbb {L}$.

### Remark 4.2

**Fig. 6:**
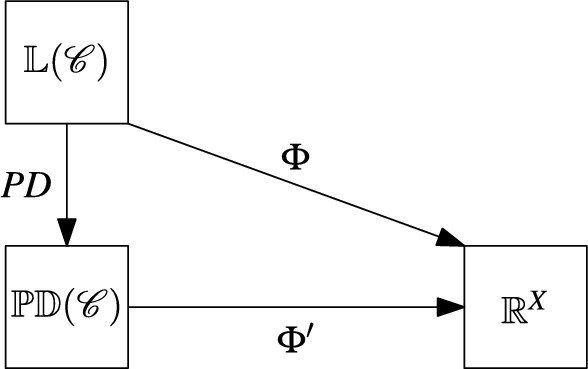
This diagram depicts the relationship between a phylogenetic diversity index $\Phi$ on $\mathbb {L}(\mathscr {C})$ for a cluster system $\mathscr {C}$ on $X$ and a phylogenetic diversity index $\Phi '$ on $\mathbb{P}\mathbb{D}(\mathscr {C})$ as described by Eq. (11)

The properties of completeness and Pareto efficiency are tightly linked. Let $\mathscr {C}$ be a cluster system on $X$ and note that $\sum _{C \in \mathscr {C}} \omega (C) = PD_{\omega }(X)$ holds for all $\omega \in \mathbb {L}(\mathscr {C})$. Therefore, every complete phylogenetic diversity index $\Phi$ on  $\mathbb {L}(\mathscr {C})$ corresponds to a phylogenetic diversity index $\Phi '$ on $\mathbb{P}\mathbb{D}(\mathscr {C})$ that satisfies Pareto efficiency, where $\Phi '$ is obtained such that the diagram in Fig. 6 commutes, that is,

11 $$\begin{aligned} \Phi '(PD_{\omega }) = \Phi (\omega ) \end{aligned}$$

for all $$\omega \in \mathbb {L}(\mathscr {C})$$.

We say that a phylogenetic diversity index $\Phi$ on a linear subspace $\mathbb {L}$ of $\mathbb {R}^{\mathscr {P}(X)}$ satisfies *group proportionality* (cf. Haake et al. 2008) if

(GP) $$(\Phi (a \cdot g_C))(x) = {\left\{ \begin{array}{ll} \frac{a}{|C|} &{}\text {if} \ x \in C\\ 0 &{}\text {if} \ x \not \in C, \end{array}\right. }$$    for all $C \in \mathscr {P}(X) {\setminus } \{\emptyset \}$ and all $a \in \mathbb {R}$

with $g_C$ the game as defined in (9). Note that a similar characterization to that given in the following theorem was established by Wicke and Steel (2020, Thm. 7) for the special case of cluster systems that form a hierarchy.

### Theorem 4.3

Let $\mathscr {C}$ be a cluster system on $X$. The Shapley value is the unique phylogenetic diversity index on $\mathbb{P}\mathbb{D}(\mathscr {C})$ that is additive and satisfies Pareto efficiency and group proportionality.

### Proof

Assume that $\Phi '$ is the Shapley value on $\mathbb{P}\mathbb{D}(\mathscr {C})$. It is known (see, e.g., Aumann 1994) that $\Phi '$ satisfies Pareto efficiency for all $\omega \in \mathbb {R}^{\mathscr {P}(X)}$ and is additive for all $\omega _1, \omega _2 \in \mathbb {R}^{\mathscr {P}(X)}$. Thus, these two properties hold, in particular, for all $\omega ,\omega _1,\omega _2 \in \mathbb{P}\mathbb{D}(\mathscr {C}) \subseteq \mathbb {R}^{\mathscr {P}(X)}$.

To establish that $\Phi '$ also satisfies group proportionality, consider $x \in X$, $C \in \mathscr {C}$ and $a \in \mathbb {R}$. We calculate the value $(\Phi '(a \cdot g_C))(x)$ using Formula (10) (similar calculations are used in the proofs of Haake et al. 2008, Thm. 4 and Coronado et al. 2018, Thm. 1):

If $x \not \in C$ we have $g_C(M)-g_C(M {\setminus } \{x\}) = 0$ for all $M \in \mathscr {P}(X)$, implying $\Phi '(a \cdot g_C))(x) = 0$, as required. So assume that $x \in C$, put $c = |C|$, $m = |M|$, and put $n = |X|$. Then, in view of the fact that only $M \in \mathscr {P}(X)$ with $M \cap C = \{x\}$ contribute to $(\Phi '(a \cdot g_C))(x)$, we have

$$\begin{aligned} (\Phi '(a \cdot g_C))(x)&= \frac{a}{n!} \cdot \sum _{m=1}^{n-c+1} (m-1)! \cdot (n-m)! \cdot {n-c \atopwithdelims ()m-1} \\&= \frac{a \cdot (n-c)! \cdot (c-1)!}{n!} \cdot \sum _{j=c-1}^{n-1} {j \atopwithdelims ()c-1}\\&= \frac{a \cdot (n-c)! \cdot (c-1)!}{n!} \cdot {n \atopwithdelims ()c} = \frac{a}{c}, \end{aligned}$$

as required, where we used the formula for the sum along a diagonal in Pascal’s triangle to obtain the first equality in the second line.

Uniqueness now follows from the fact that, in view of the proof of Lemma 4.1, $\mathbb{P}\mathbb{D}(\mathscr {C})$ is the linear span of $\{g_C: C \in \mathscr {C}\}$. $\square$

Interestingly, as shown by Fuchs and Jin (2015), the vector in $\mathbb {R}^X$ that results from computing the Shapley value on the game $PD$ obtained from an edge-weighted rooted phylogenetic tree always coincides with the vector that we obtain by computing the fair proportion index on the rooted phylogenetic tree. In fact, this is a particular instance of (11). The following corollary of Theorem 4.3 makes this more precise.

### Corollary 4.4

Let $\mathscr {C}$ be a cluster system on $X$, $\Phi$ be the fair proportion index on $\mathbb {L}(\mathscr {C})$, and $\Phi '$ be the Shapley value on $\mathbb{P}\mathbb{D}(\mathscr {C})$. Then

$$\begin{aligned}\Phi (\omega ) = \Phi '(\sum _{C \in \mathscr {C}} \omega (C) \cdot g_C) = \Phi '(PD_{\omega })\end{aligned}$$

holds for all $\omega \in \mathbb {L}(\mathscr {C})$.

### Proof

This follows immediately from the definition of the fair proportion index together with the fact that, by Theorem 4.3, the Shapley value is additive and satisfies group proportionality. $\square$

It is remarked in the discussion by Coronado et al. (2018) that Corollary 4.4 can also be derived using arguments based on so-called phylogenetic networks (for more on the connection between such networks and diversity indices see Sect. 8). Moreover, the fact that the Shapley value on $\mathbb{P}\mathbb{D}(\mathscr {C})$ satisfies Pareto efficiency means that it apportions the phylogenetic diversity of *X* among the elements of *X*. In view of Corollary 4.4 this then also holds for the fair proportion index on $\mathbb {L}(\mathscr {C})$ and, in view of Remark 4.2, this corresponds to the fact that the fair proportion index is complete, as can be seen in the example in Fig. 3a:

$$\begin{aligned}PD_{\omega }(X) = \sum _{C \in \mathscr {C}} \omega (C) = 25 = \sum _{x \in X} (FP(\omega ))(x).\end{aligned}$$

## An Affine and Projective Framework for Phylogenetic Diversity Indices

As mentioned in the introduction, the notion of phylogenetic diversity indices has also been considered on unrooted phylogenetic trees (Haake et al. 2008; Wicke and Steel 2020) and, just as rooted phylogenetic trees can be encoded by a collection of clusters, unrooted phylogenetic trees on a set $X$ of species can be encoded by a collection $\mathscr {S}$ of bipartitions, or splits, of *X* (see, e.g., Steel 2016, Ch. 2). In the area of phylogenetic combinatorics, the interplay between collections of clusters and collections of splits has been studied in terms of affine and projective models of clustering, respectively, in analogy with the interplay between affine and projective geometry in classical geometry (Dress 2012, p. 207; see also Dress 1997). One of the key ideas that we will exploit from this theory is that we can map a collection $\mathscr {S}$ of splits of $X$ in a natural way to a cluster system $\mathscr {C}(\mathscr {S})$ on $X$ (defined in (13) below) and, in this way, derive split-based indices from cluster-based indices. In this section, we will make this more precise, and illustrate the resulting framework using the fair proportion index and the Shapely value as examples.

First, we formally define the concepts mentioned above. A *split* $S$ of $X$ is a bipartition of $X$ into two non-empty subsets $A$ and $B$, that is, $A \cup B = X$ and $A \cap B = \emptyset$. We denote such a split as an unordered pair $A|B = B|A$. A *split system* $\mathscr {S}$ on $X$ is a non-empty set of splits of $X$. By $\mathscr {S}(X)$ we denote the set of all splits of $X$ and, for a split system $\mathscr {S} \subseteq \mathscr {S}(X)$, we denote by $\mathbb {L}(\mathscr {S})$ the set of all weightings $\lambda : \mathscr {S}(X) \rightarrow \mathbb {R}$ with $\lambda (S) = 0$ for all $S \in \mathscr {S}(X) {\setminus } \mathscr {S}$. In addition, we denote by $\mathbb{P}\mathbb{D}(\mathscr {S})$ the set of all weightings $PD: \mathscr {P}(X) \rightarrow \mathbb {R}$ that can be written as

12 $$\begin{aligned} PD(M) = PD_{\lambda }(M) = \sum _{A|B \in \mathscr {S}: \ A \cap M \ne \emptyset , B \cap M \ne \emptyset } \lambda (A|B) \end{aligned}$$

for some $\lambda \in \mathbb {L}(\mathscr {S})$. The value $PD_{\lambda }(M)$ is usually called the *phylogenetic diversity* of $M$ with respect to the weighting $\lambda$ of the splits in $\mathscr {S}$ (see, e.g., Spillner et al. 2008).

**Fig. 7:**
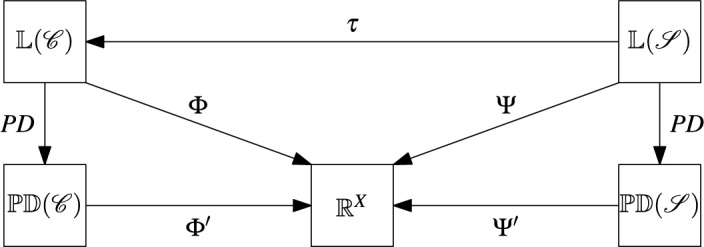
A diagram of the various maps we consider to study relationships between phylogenetic diversity indices. The left part of the diagram we have already seen in Fig. 6. In analogy to this, the right part of the diagram depicts phylogenetic diversity indices $\Psi$ and $\Psi '$ on $\mathbb {L}(\mathscr {S})$ and $\mathbb{P}\mathbb{D}(\mathscr {S})$, respectively, where $\mathscr {S}$ is a split system on $X$. Finally $\tau$ associates with each weighting $\lambda$ of the splits in $\mathscr {S}$ a weighting $\omega = \tau (\lambda )$ of the clusters in a cluster system $\mathscr {C} = \mathscr {C}(\mathscr {S})$ that arises from $\mathscr {S}$ by (13)

Figure 7 gives an overview of the various spaces we shall consider and the maps between them. In addition to the maps already introduced in Fig. 6 in Sect. 4, we also consider, for split systems $\mathscr {S}$ on $X$, maps $\tau$ from $\mathbb {L}(\mathscr {S})$ to $\mathbb {L}(\mathscr {C})$ where $\mathscr {C}$ is the cluster system

13 $$\begin{aligned} \mathscr {C}(\mathscr {S}) = \bigcup _{S \in \mathscr {S}} S \end{aligned}$$

on $X$ mentioned above. In particular, we are interested in maps $\tau$ for which various parts of the diagram in Fig. 7 commute.

As an illustration of this setup, we now revisit the relationship between the fair proportion index and the Shapely value. Let $\mathscr {S}$ be a split system on $X$. Then the *Shapley value* on $\mathbb{P}\mathbb{D}(\mathscr {S})$ is defined as in (10). Equivalently, as shown by Haake et al. (2008) for trees and by Volkmann et al. (2014) for split systems in general, the Shapley value on $\mathbb{P}\mathbb{D}(\mathscr {S})$ can also be computed as

14 $$\begin{aligned} (SV(PD_{\lambda }))(x) = \sum _{A|B \in \mathscr {S}: \ x \in A} \frac{|B|}{|X| \cdot |A|} \cdot \lambda (A|B). \end{aligned}$$

for all $\lambda \in \mathbb {L}(\mathscr {S})$ and all $x \in X$.

**Fig. 8:**
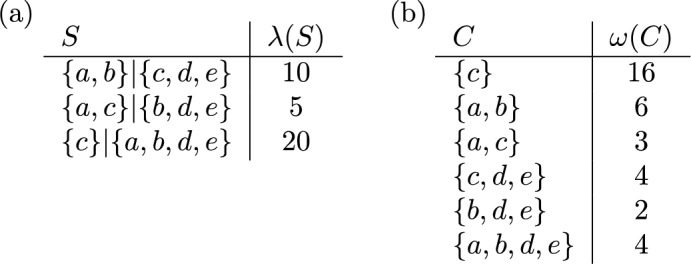
**a** A split system $\mathscr {S}$ on $X=\{a,b,c,d,e\}$ with weighting $\lambda$. **b** The associated cluster system $\mathscr {C}(\mathscr {S})$ on $X$ as defined in (13) and the weighting $\omega = \tau (\lambda )$ as defined in (15)

Now consider the map $\tau : \mathbb {L}(\mathscr {S}) \rightarrow \mathbb {L}(\mathscr {C}(\mathscr {S}))$ defined by putting, for $\lambda \in \mathbb {L}(\mathscr {S})$,

15 $$\begin{aligned} (\tau (\lambda ))(A) = \frac{|B|}{|X|} \cdot \lambda (A|B) \ \text { and } \ (\tau (\lambda ))(B) = \frac{|A|}{|X|} \cdot \lambda (A|B) \end{aligned}$$

for all $A,B \in \mathscr {C}(\mathscr {S})$ such that $A|B$ is a split in $\mathscr {S}$. For example, consider the split system $\mathscr {S}$ with weighting $\lambda$ in Fig. 8a. Using Formula (14), we obtain $SV(a) = \frac{11}{2}$ in this example and we also have $FP(a) = \frac{11}{2}$ for the fair proportion index as defined in (4) applied to the cluster system $\mathscr {C}(\mathscr {S})$ with weighting $\omega = \tau (\lambda )$. We conclude this section by showing that this is not a coincidence.

### Theorem 5.1

Let $\mathscr {S}$ be a split system on $X$, $\Phi$ be the fair proportion index on $\mathbb {L}(\mathscr {C}(\mathscr {S}))$ and $\Psi '$ be the Shapley value on $\mathbb{P}\mathbb{D}(\mathscr {S})$. If $\tau$ is as defined in (15), then

16 $$\begin{aligned} \Phi (\tau (\lambda )) = \Psi '(PD_{\lambda }) \end{aligned}$$

for all $$\lambda \in \mathbb {L}(\mathscr {S})$$.

### Proof

Let $\lambda \in \mathbb {L}(\mathscr {S})$ and put $\omega = \tau (\lambda )$. Since the maps $\Phi$, $\tau$, $\Psi '$ and $PD$ are all linear, it suffices to show Eq. (16) for the case that one element of ${{\mathscr {S}}}$, say $S=A|B$ has weight 1 (i.e. $\lambda (A|B)=1$), whereas $\lambda (S')=0$ for all $S' \ne S$. Then we have $\omega (A) = |B|/|X|$, $\omega (B)=|A|/|X|$, and $\omega (C)=0$ for all $C \in {{\mathscr {C}}}({{\mathscr {S}}})$ with $C \ne A,B$. Now let $x \in X$, and assume without loss of generality that $x \in A$. Then,

$$\begin{aligned} (\Phi (\omega ))(x) = \sum \limits _{C \in {{\mathscr {C}}}({{\mathscr {S}}}): \ x \in C} \frac{\omega (C)}{|C|} = \frac{\omega (A)}{|A|} = \frac{\frac{|B|}{|X|}}{|A|} = \frac{|B|}{|X| \cdot |A|}. \end{aligned}$$

On the other hand, in view of (14) we have

$$\begin{aligned} (\Psi '(PD_{\lambda }))(x) = \sum \limits _{A'|B' \in {{\mathscr {S}}}: \ x \in A'} \frac{|B'|}{|X| \cdot |A'|} \lambda (S) = \frac{|B|}{|X| \cdot |A|} \end{aligned}$$

as well. This completes the proof. $\square$

## Complete Diversity Indices

In this section we shall consider Fig. 7 once again, considering an alternative definition for the map $\tau$ that can be used to translate, for any split system $\mathscr {S}$ on $X$, the property of completeness from a cluster-based index $\Phi$ on $\mathbb {L}(\mathscr {C}(\mathscr {S}))$ to an associated split-based index $\Psi = \Psi _{\tau }(\Phi )$ on $\mathbb {L}(\mathscr {S})$. In particular, we will see that this immediately implies the completeness of the fair proportion index on unrooted phylogenetic trees that was established by Wicke and Steel (2020) (for example, see Fig. 1b in the introduction). In addition, we illustrate the application of these considerations to a generalization of the so-called equal splits index that appears in Wicke and Steel (2020).

We begin by proving a result concerning completeness. Let $\mathscr {S}$ be a split system on $X$. A phylogenetic diversity index $\Psi$ on $\mathbb {L}(\mathscr {S})$ is *complete* if

(C’): $\sum _{x \in X} (\Psi (\lambda ))(x) = \sum _{S \in \mathscr {S}} \lambda (S)$ holds for all $\lambda \in \mathbb {L}(\mathscr {S})$.

Define the map $\tau :\mathbb {L}(\mathscr {S}) \rightarrow \mathbb {L}(\mathscr {C}(\mathscr {S}))$ by putting

17 $$\begin{aligned} (\tau (\lambda ))(C) = \frac{1}{2} \cdot \lambda (C|(X-C)) \end{aligned}$$

for all $C \in \mathscr {C}(\mathscr {S})$. The basic idea is to distribute the weight $\lambda (S)$ of a split $S=A|B \in \mathscr {S}$ evenly on the two corresponding clusters $A,B \in \mathscr {C}(\mathscr {S})$. The following results, however, also hold if the weight is distributed non-evenly, that is, when putting $(\tau (\lambda ))(A) = p \cdot \lambda (A\vert B)$ and $(\tau (\lambda ))(B) = (1-p) \cdot \lambda (A\vert B)$ for some $0< p < 1$ with $p \ne \frac{1}{2}$.

With the map $\tau$ defined in (17), we obtain, for a phylogenetic diversity index $\Phi$ on $\mathbb {L}(\mathscr {C}(\mathscr {S}))$, the phylogenetic diversity index $\Psi = \Psi _{\tau }(\Phi )$ on $\mathbb {L}(\mathscr {S})$ by putting $\Psi (\lambda ) = \Phi (\tau (\lambda ))$ for all $\lambda \in \mathbb {L}(\mathscr {S})$.

### Theorem 6.1

Let $\mathscr {S}$ be a split system on $X$ and $\Phi$ a complete linear phylogenetic diversity index on $\mathbb {L}(\mathscr {C}(\mathscr {S}))$. If $\tau$ is as defined in (17), then $\Psi _{\tau }(\Phi )$ is a complete linear phylogenetic diversity index on $\mathbb {L}(\mathscr {S})$.

### Proof

Let $\Phi$ be a complete linear phylogenetic diversity index on $\mathbb {L}(\mathscr {C}(\mathscr {S}))$. We first show that $\Psi _{\tau }$ is linear. Let $\lambda _1, \lambda _2 \in \mathbb {L}(\mathscr {S})$ and $a \in \mathbb {R}$. Then, noting that $\tau$ is linear, we have

$$\begin{aligned} (\Psi _{\tau }(\Phi ))(a \cdot \lambda _1 + \lambda _2)&= \Phi (\tau (a \cdot \lambda _1 + \lambda _2)) = \Phi (a \cdot \tau (\lambda _1) + \tau (\lambda _2)) \\&= a \cdot \Phi (\tau (\lambda _1)) + \Phi (\tau (\lambda _2)) = a \cdot (\Psi _{\tau }(\Phi ))(\lambda _1) + (\Psi _{\tau }(\Phi ))(\lambda _2), \end{aligned}$$

as required.

It remains to show that $\Psi _{\tau }$ is complete. Let $\lambda \in \mathbb {L}(\mathscr {S})$. Then we have

$$\begin{aligned} \sum _{x \in X} ((\Psi _{\tau }(\Phi ))(\lambda ))(x)&= \sum _{x \in X} (\Phi (\tau (\lambda )))(x) = \sum _{C \in \mathscr {C}(\mathscr {S})} (\tau (\lambda ))(C)\\&= \sum _{C \in \mathscr {C}(\mathscr {S})} \frac{1}{2} \cdot \lambda (C|X-C) = \sum _{S \in \mathscr {S}} \lambda (S), \end{aligned}$$

as required. $$\square $$

The following Corollary 6.2 includes, as a special case, the completeness of the fair proportion index on unrooted phylogenetic trees that was established by Wicke and Steel (2020, Thm. 10). To see this, it suffices to consider, for an unrooted phylogenetic tree on $X$, the split system consisting of those splits of $X$ that can be obtained by removing an edge from the tree.

### Corollary 6.2

Let $\mathscr {S}$ be a split system on $X$ and $\Phi$ be the fair proportion index on $\mathbb {L}(\mathscr {C}(\mathscr {S}))$. If $\tau$ is as defined in (17), then $\Psi _{\tau }(\Phi )$ is a complete linear phylogenetic diversity index on $\mathbb {L}(\mathscr {S})$ and we have

18 $$\begin{aligned} ((\Psi _{\tau }(\Phi ))(\lambda ))(x) = \sum _{A|B \in \mathscr {S}: \ x \in A} \frac{\lambda (S)}{2 \cdot |A|} \end{aligned}$$

for all $\lambda \in \mathbb {L}(\mathscr {S})$ and all $x \in X$.

### Proof

In view of Lemma 2.1, Theorem 6.1 implies that $\Psi _{\tau }(\Phi )$ is a complete linear phylogenetic diversity index on $\mathbb {L}(\mathscr {S})$. Moreover, (18) follows from (4), (13), and (17). $\square$

**Fig. 9:**
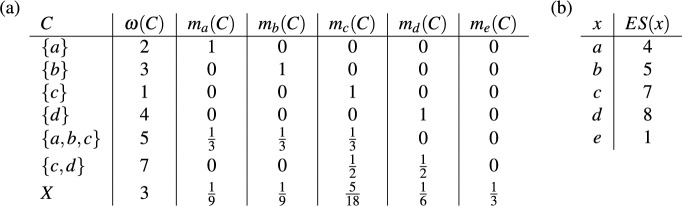
**a** A cluster system $\mathscr {C}$ on $X = \{a,b,c,d,e\}$ with weighting $\omega$ and the quantities $m_x(C)$ as defined for all $x \in X$ and $C \in \mathscr {C}$ in (19). **b** The equal splits index $ES(x)$ for all $x \in X$ obtained from $\mathscr {C}$ and $\omega$ by (20)

We now turn our attention to a generalization of the *equal splits* index, a phylogenetic diversity index that was introduced in the setting of rooted phylogenetic trees by Redding and Mooers (2006). We first define our generalization for cluster systems ${{\mathscr {C}}}$ on *X*. For all $C \in \mathscr {C}$, let *cl*(*C*) denote the set of those $x \in C$ that are not contained in any cluster $C' \in ch(C)$. Then put

19 $$\begin{aligned} m_x(C) = {\left\{ \begin{array}{ll} 0 &{}\text {if} \ x \notin C, \\ \frac{1}{|ch(C)|+|cl(C)|} &{}\text {if} \ x \in cl(C), \\ \sum \limits _{C' \in ch(C)} \frac{m_x(C')}{|ch(C)| + |cl(C)|} &{}\text {otherwise,} \end{array}\right. } \end{aligned}$$

for all $x \in X$ and all $C \in \mathscr {C}$. Note that $m_x(C)=1/|C|$ if $x \in C$ and $ch(C)=\emptyset$ (as in this case $|ch(C)|=0$ and $|cl(C)|=|C|$). Also note that $m_x(C)$ is defined recursively. In particular, when computing $m_x(C)$ in the third case it is assumed that $m_x(C')$ for each $C' \in {{\mathscr {C}}}$ with $C' \subsetneq C$ has been computed already. The equal splits index is then defined by putting

20 $$\begin{aligned} (ES(\omega ))(x) = \sum _{C \in {{\mathscr {C}}}} m_x(C) \cdot \omega (C) \end{aligned}$$

for all $\omega \in \mathbb {L}({{\mathscr {C}}})$ and all $x \in X$. As an example, consider the cluster system $\mathscr {C}$ with weighting $\omega$ in Fig. 9a. For the cluster $C=X$ we have $ch(X) = \{\{a,b,c\},\{c,d\}\}$ and $cl(X) = \{e\}$, which yields, by (19), $m_e(X) = \frac{1}{3}$. The resulting values of the equal splits index are given in Fig. 9b.

The equal splits index on $\mathbb {L}(\mathscr {C})$ is linear with the corresponding $|\mathscr {C}| \times |X|$-matrix $\Gamma$ in Eq. (5) having the entries $\gamma _{(C,x)} = m_x(C)$. Moreover, as can be seen in the example in Fig. 9a, the sum of the entries in each row of $\Gamma$ equals 1. The next theorem establishes that this is always the case.

### Theorem 6.3

For all cluster systems $\mathscr {C}$ on $X$ the equal splits index is a complete linear phylogenetic diversity index on $\mathbb {L}(\mathscr {C})$.

### Proof

Let ${{\mathscr {C}}}$ be a cluster system on $X$. We already noted above that the equal splits index is linear. Thus, it remains to establish that the equal splits index is complete. More specifically, it suffices to show that

21 $$\begin{aligned} \sum _{x \in X} m_x(C)=1 \end{aligned}$$

for all $C \in {{\mathscr {C}}}$. To this end, put $desc(C) = |\bigcup _{C' \in ch(C)} C' |$ for all $C \in {{\mathscr {C}}}$, that is, *desc*(*C*) equals the total number of elements in $X$ contained in the clusters in *ch*(*C*). We show (21) by induction on *desc*(*C*).

For the base case $desc(C)=0$ we have $ch(C) = \emptyset$ and thus $cl(C) = C$, implying $|cl(C)|=|C|$ and

$$\begin{aligned}\sum \limits _{x \in X} m_x(C) = \sum \limits _{x \in C} m_x(C) + \sum \limits _{x \in X \setminus C} m_x(C) = |C| \cdot \frac{1}{|C|} + 0 = 1,\end{aligned}$$

as required.

Next assume $desc(C) > 0$. By the definition of *ch*(*C*), we have $desc(C') < desc(C)$ for all $C' \in ch(C)$. Thus,

$$\begin{aligned} \sum \limits _{x \in X} m_x(C)&= \sum \limits _{x \in X \setminus C} m_x(C) + \sum \limits _{x \in cl(C)} m_x(C) + \sum \limits _{x \in C \setminus cl(C)} m_x(C) \\&= 0 + \frac{|cl(C)|}{|ch(C)|+|cl(C)|} + \sum \limits _{x \in C \setminus cl(C)} \ \sum \limits _{C' \in ch(C)} \frac{m_x(C')}{|ch(C)| + |cl(C)|}\\&= \frac{|cl(C)|}{|ch(C)|+|cl(C)|} + \sum \limits _{C' \in ch(C)} \ \sum \limits _{x \in C \setminus cl(C)} \frac{m_x(C')}{|ch(C)| + |cl(C)|}\\&= \frac{|cl(C)|}{|ch(C)|+|cl(C)|} + \sum \limits _{C' \in ch(C)} \ \sum \limits _{x \in X} \frac{m_x(C')}{|ch(C)| + |cl(C)|} \\&= \frac{|cl(C)|}{|ch(C)|+|cl(C)|} + |ch(C)| \cdot \frac{1}{|ch(C)| + |cl(C)|}\\&= 1, \end{aligned}$$

where the equality in the fourth line holds in view of the fact that $m_x(C') = 0$ for all $x \in X {\setminus } (C {\setminus } cl(C))$ and for all $C' \in ch(C)$, and the equality in the fifth line holds by induction. $\square$

Our final result in this section, which is an immediate consequence of Theorem 6.1 and Theorem 6.3, summarizes how we obtain, via the map $\tau$ defined in (17), a complete linear split-based phylogenetic diversity index from the cluster-based equal splits index.

### Corollary 6.4

Let $\mathscr {S}$ be a split system on $X$ and $\Phi$ be the equal splits index on $\mathbb {L}(\mathscr {C}(\mathscr {S}))$. If $\tau$ is as defined in (17), then $\Psi _{\tau }(\Phi )$ is a complete linear phylogenetic diversity index on $\mathbb {L}(\mathscr {S})$.

We conclude this section coming back to the biological example in Fig. 2a and compute the phylogenetic diversity index $\Psi _{\tau } = \Psi _{\tau }(\Phi )$ from Corollary 6.4 for this example. From the split system $\mathscr {S}$ on $X=\{a,f,h,m,r,s\}$ with weighting $\lambda$ given in Fig. 12 in the Appendix we first compute the cluster system $\mathscr {C} = \mathscr {C}(\mathscr {S})$ on $X$ with weighting $\omega = \tau (\lambda )$ (in Fig. 13 in the Appendix we present the Hasse diagram for the 20 clusters in $\mathscr {C}$, where the weight $\omega (C)$ obtained by (17) is given below each cluster $C$ in the diagram). Then we compute the matrix $\Gamma = \Gamma _{\Phi }$ for the equal splits index $\Phi$ on $\mathbb {L}(\mathscr {C})$ (see Fig. 14 in the Appendix) from which we obtain the values of the phylogenetic diversity index $\Psi _{\tau }$ given in Fig. 2b. For comparison purposes, we also compute the Shapley value $SV$ as defined in (14) for the split system $\mathscr {S}$ on $X$ with weighting $\lambda$ using the program SplitsTreeCE (Huson and Bryant 2005).

As can be seen in Fig. 2b, the sum of the values of the index $\Psi _{\tau }$ yields the total weight $1.980$ of all splits in $\mathscr {S}$, as it should be for a complete phylogenetic diversity index. The ranking of the six populations given by $SV$ is the same as the ranking given by $FP_u$ computed in the introduction. The ranking given by $\Psi _{\tau }$ slightly deviates from it but also ranks populations $s$ and $m$ at the top and population $f$ at the bottom.

The fact that the network in Fig. 2a is not a tree implies that using a tree-based phylogenetic diversity index necessarily involves a (potentially arbitrary) decision which of the splits in $\mathscr {S}$ are used to compute the tree-based index. More formally, we would first need to restrict to some subset $\mathscr {S}' \subseteq \mathscr {S}$ such that any two splits $A|B, C|D \in \mathscr {S}'$ are *compatible*, that is, at least one of the intersections $A \cap C$, $A \cap D$, $B \cap C$, and $B \cap D$ is empty. A collection of pairwise compatible splits is called a *compatible* split system. Intuitively, compatible split systems correspond to unrooted phylogenetic trees.

To illustrate that the choice of a compatible subset of $\mathscr {S}$ really has an impact on the ranking of the six owl populations, we consider two compatible subsets $\mathscr {S}_1'$ and $\mathscr {S}_2'$ that are maximal with respect to set inclusion. Using the index $\Psi _{\tau }$, we obtain the ranking (from highest to lowest) *s*, *m*, *h*, *r*, *a*, *f* based on $\mathscr {S}_1'$ and the ranking *s*, *m*, *a*, *h*, *r*, *f* based on $\mathscr {S}_2'$ (for details see Figs. 15 and 16 in the Appendix). Clearly, these two rankings are different, and in fact they also differ from the ranking obtained when considering all splits in ${{\mathscr {S}}}$ (cf. Fig. 2b). Interestingly, in this example, the ranking given in Fig. 2b for $FP_u$ and *SV* does not change when restricting to *any* maximal compatible subset of $\mathscr {S}$. This could be due to the fact that the *trivial* splits in $\mathscr {S}$ (i.e. splits *A*|*B* with $|A|=1$ or $|B|=1$) carry more weight than the *non-trivial* splits, and both $FP_u$ and *SV* are less heavily influenced by the non-trivial splits than $\Psi _{\tau }$. In future work, it could be interesting to further investigate the differences in rankings obtained from these and other split-based diversity indices.

## A Generalization of the Pauplin Index

In Wicke and Steel 2020, (Sec. 5.2) a phylogenetic diversity index for unrooted phylogenetic trees is introduced that is related to the formula for the total weight of the edges given by Pauplin (2000). In this section, we describe how the viewpoint suggested by our framework leads to a new family of split-based phylogenetic diversity indices.

Let $n = |X| \ge 3$ and $\theta = x_0,x_1,\dots ,x_{n-1}$ be an ordering of the elements in $X$. Define the split system

$$\begin{aligned} \mathscr {S}_{\theta } = \{\{x_i,\dots ,x_j\}|X \setminus \{x_i,\dots ,x_j\}: 0 \le i \le j < n-1\}. \end{aligned}$$

A split system $\mathscr {S}$ on $X$ is *circular* if there exists an ordering $\theta$ of the elements in $X$ such that $\mathscr {S} \subseteq \mathscr {S}_{\theta }$. If $\mathscr {S} = \mathscr {S}_{\theta }$ for some ordering $\theta$ of the elements in $X$ we call $\mathscr {S}$ a *full* circular split system on $X$. Clearly, a circular split system contains at most $$\left( {\begin{array}{c}n\\ 2\end{array}}\right) $$ splits. Moreover, every compatible split system is circular (Bandelt and Dress 1992). Volkmann et al. (2014) considered the Shapley value and another phylogenetic diversity index on weighted circular split systems (see also the more recent work by Abhari et al. (2024)).

Let $$\left( {\begin{array}{c}X\\ 2\end{array}}\right) $$ denote the set of all 2-element subsets of $X$. For a circular split system $\mathscr {S}$ on $X$ we consider the $$|\mathscr {S}| \times \left( {\begin{array}{c}n\\ 2\end{array}}\right) $$-matrix $M(\mathscr {S})$ whose rows correspond to the splits in $\mathscr {S}$ and whose columns correspond to the 2-element subsets in $$\left( {\begin{array}{c}X\\ 2\end{array}}\right) $$. The entry of $M = M(\mathscr {S})$ corresponding to $S=A|B \in \mathscr {S}$ and $$\{x,y\} \in \left( {\begin{array}{c}X\\ 2\end{array}}\right) $$ is defined as

$$\begin{aligned} m_{(S,\{x,y\})} = {\left\{ \begin{array}{ll} 1 &{}\text {if} \ |\{x,y\} \cap A| = |\{x,y\} \cap B| = 1\\ 0 &{}\text {otherwise}. \end{array}\right. } \end{aligned}$$

The matrix $M(\mathscr {S})$ describes how a weighting $\lambda \in \mathbb {L}(\mathscr {S})$ gives rise to pairwise distances between the elements in $X$:

22 $$\begin{aligned} d_{\lambda }(\{x,y\}) = \sum _{S \in \mathscr {S}} \lambda (S) \cdot m_{(S,\{x,y\})}, \end{aligned}$$

or, more compactly, $d_{\lambda } = \lambda \cdot M$, where $\lambda$ is viewed as a row vector with $|\mathscr {S}|$ entries and $d_{\lambda }$ as a row vector with $$\left( {\begin{array}{c}n\\ 2\end{array}}\right) $$ entries. The rows of $M(\mathscr {S})$ are linearly independent (Bandelt and Dress 1992). Thus, every weighting $\lambda$ yields a distinct $d_{\lambda }$.

Since the matrix $M(\mathscr {S})$ has full rank, there exists an $$\left( {\begin{array}{c}n\\ 2\end{array}}\right) \times |\mathscr {S}|$$-matrix $R$ that is *right inverse* to $M(\mathscr {S})$, that is, $M(\mathscr {S}) \cdot R$ yields the $|\mathscr {S}| \times |\mathscr {S}|$-identity matrix. The matrix $R$ need not be unique, however. Any such matrix corresponds to a linear estimator of a weighting of the splits in $\mathscr {S}$ from pairwise distances between the elements in $X$ (see e.g. Pardi and Gascuel (2012) for a discussion of such estimators for unrooted phylogenetic trees). Fixing such a matrix $R$, we define a phylogenetic diversity index $\Psi _R$ on $\mathbb {L}(\mathscr {S})$ by putting

$$\begin{aligned} (\Psi _R(\lambda ))(x) = \frac{1}{2} \cdot \sum _{y \in X \setminus \{x\}} \ \sum _{S \in \mathscr {S}} d_{\lambda }(\{x,y\}) \cdot r_{(\{x,y\},S)} \end{aligned}$$

for all $x \in X$ and all $\lambda \in \mathbb {L}(\mathscr {S})$. In view of (22), we have $\Psi _R(\lambda ) = \lambda \cdot \Gamma _R$ for the $|\mathscr {S}| \times |X|$-matrix $\Gamma = \Gamma _{\Psi _R}$ whose entries are

$$\begin{aligned} \gamma _{(S,x)} = \frac{1}{2} \cdot \sum _{y \in X \setminus \{x\}} \ \sum _{S' \in \mathscr {S}} m_{(S,\{x,y\})} \cdot r_{(\{x,y\},S')}. \end{aligned}$$

Moreover, since $R$ is right inverse to $M(\mathscr {S})$, we have

$$\begin{aligned} \sum _{x \in X} \gamma _{(S,x)}{} & {} = \frac{1}{2} \cdot \sum _{x \in X} \ \sum _{y \in X \setminus \{x\}} \ \sum _{S' \in \mathscr {S}} m_{(S,\{x,y\})} \cdot r_{(\{x,y\},S')} \\{} & {} = \sum _{\{x,y\} \in \left( {\begin{array}{c}X\\ 2\end{array}}\right) } \ \sum _{S' \in \mathscr {S}} m_{(S,\{x,y\})} \cdot r_{(\{x,y\},S')} = 1 \end{aligned}$$

for all $S \in \mathscr {S}$. The next lemma summarizes these basic facts about $\Psi _R$.

### Lemma 7.1

Let $\mathscr {S}$ be a circular split system on $X$ with $|X| \ge 3$. Then, for every matrix $R$ that is right inverse to the matrix $M(\mathscr {S})$, $\Psi _R$ is a complete linear phylogenetic diversity index on $\mathbb {L}(\mathscr {S})$.

**Fig. 10:**
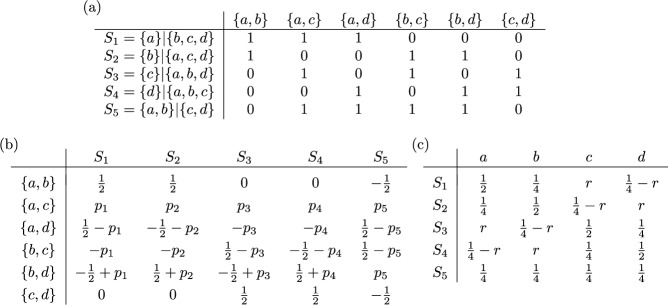
**a** The matrix $M(\mathscr {S})$ for the split system $\mathscr {S} = \{S_1,\dots ,S_5\}$ on $X=\{a,b,c,d\}$. **b** The matrices $R$ that are right inverse to $M(\mathscr {S})$. **c** The matrix $\Gamma _{\Psi _R}$ for the phylogenetic diversity index $\Psi _R$ on $\mathbb {L}(\mathscr {S})$

Consider, as an example, the split system $\mathscr {S} = \{S_1,\dots ,S_5\}$ on $X = \{a,b,c,d\}$, for which the matrix $M(\mathscr {S})$ is given in Fig. 10a. The split system $\mathscr {S}$ is compatible and, thus, circular. There are infinitely many matrices $R$ that are right inverse to the matrix $M(\mathscr {S})$ and they can be described by five parameters $p_1,\dots ,p_5 \in \mathbb {R}$ as shown in Fig. 10b. The matrix $\Gamma _{\Psi _R}$ for the resulting complete linear phylogenetic diversity index $\Psi _R$ is given in Fig. 10c. This index has only a single parameter $r \in \mathbb {R}$ with $r = \frac{1}{2}(p_1+\dots +p_5)$. If all entries in $\Gamma _{\Psi _R}$ are required to be non-negative, we need to restrict this parameter to $0 \le r \le \frac{1}{4}$. The Pauplin index in Wicke and Steel (2020, Sec. 5.2) corresponds to $r=\frac{1}{8}$.

We now present the main result of this section which focuses on full circular split systems.

### Theorem 7.2

Let $\mathscr {S}$ be a full circular split system on $X$ with $\mathscr {S} = \mathscr {S}_{\theta }$ for the ordering $\theta =x_0,x_1,\dots ,x_{n-1}$ of $X$. Then the matrix $M(\mathscr {S})$ has a unique right inverse matrix $R$ and the matrix $\Gamma =\Gamma _{\Psi _R}$ for the complete linear phylogenetic diversity index $\Psi _R$ on $\mathbb {L}(\mathscr {S})$ has the entryfor the split $S = \{x_i,\dots ,x_j\}|X{\setminus }\{x_i,\dots ,x_j\} \in \mathscr {S}$ and the element $x=x_k \in X$.

### Proof

Since $\mathscr {S}$ is a full circular split system, the matrix $M=M(\mathscr {S})$ is a square matrix. Hence, $M$ has a unique right inverse matrix $R$ which is just the usual inverse matrix of $M$. Moreover, as shown by Chepoi and Fichet (1998), the matrix $R$ has the entry

23 $$\begin{aligned} r_{(\{y,z\},S')} = {\left\{ \begin{array}{ll} \frac{1}{2} &{}\text {if} \ \{y,z\} = \{a,(b+1) \ \textrm{mod} \ n\} \ \text {or} \ \{y,z\} = \{(a-1) \ \textrm{mod} \ n,b\}\\ -\frac{1}{2} &{}\text {if} \ \{y,z\} = \{a,b\} \ \text {or} \ \{y,z\} = \{(a-1) \ \textrm{mod} \ n,(b+1) \ \textrm{mod} \ n\}\\ 0 &{}\text {otherwise} \end{array}\right. }\nonumber \\ \end{aligned}$$

for $$\{y,z\} \in \left( {\begin{array}{c}X\\ 2\end{array}}\right) $$ and $$S' = \{x_a,\dots ,x_b\}|X {\setminus } \{x_a,\dots ,x_b\} \in \mathscr {S}$$.

Consider the split $S = \{x_i,\dots ,x_j\}|X {\setminus } \{x_i,\dots ,x_j\} \in \mathscr {S}$ and the element $x=x_k \in X$. By symmetry, it suffices to consider the following three cases.

Case 1: $i< k < j$. Consider $y \in X {\setminus } \{x\}$ with $m_{(S,\{x,y\})} = 1$. Then, by the definition of the matrix $M$, we have $y \in X {\setminus } \{x_i,\dots ,x_j\}$. This implies, in view of (23), that there exist precisely two splits $S' \in \mathscr {S}$ with $r_{(\{x,y\},S')} = \frac{1}{2}$ and precisely two splits $S' \in \mathscr {S}$ with $r_{(\{x,y\},S')} = -\frac{1}{2}$. Hence, we have

$$\begin{aligned} \sum _{S' \in \mathscr {S}} m_{(S,\{x,y\})} \cdot r_{(\{x,y\},S')} = 0, \end{aligned}$$

implying that $\gamma _{(S,x)} = \frac{1}{2} \cdot 0 = 0$, as required.

Case 2: $i = k < j$. Consider again $y \in X {\setminus } \{x\}$ with $m_{(S,\{x,y\})} = 1$. If $y \ne x_{(i-1) \ \textrm{mod} \ n}$, we have $\sum _{S' \in \mathscr {S}} m_{(S,\{x,y\})} \cdot r_{(\{x,y\},S')} = 0$ using the same argument as in Case 1. Otherwise, there exist precisely two splits $S' \in \mathscr {S}$ with $r_{(\{x,y\},S')} = \frac{1}{2}$ but only one split $S' \in \mathscr {S}$ with $r_{(\{x,y\},S')} = -\frac{1}{2}$, implying

$$\begin{aligned} \sum _{S' \in \mathscr {S}} m_{(S,\{x,y\})} \cdot r_{(\{x,y\},S')} = \frac{1}{2} \end{aligned}$$

and, thus, $\gamma _{(S,x)} = \frac{1}{2} \cdot \frac{1}{2} = \frac{1}{4}$, as required.

Case 3: $i = k = j$. Then we have $m_{(S,\{x,y\})} = 1$ for all $y \in X {\setminus } \{x\}$. If $y \not \in \{x_{(k-1) \ \textrm{mod} \ n},x_{(k+1) \ \textrm{mod} \ n}\}$ we again have $\sum _{S' \in \mathscr {S}} m_{(S,\{x,y\})} \cdot r_{(\{x,y\},S')} = 0$ by the argument used in Case 1. Otherwise, we have $\sum _{S' \in \mathscr {S}} m_{(S,\{x,y\})} \cdot r_{(\{x,y\},S')} = \frac{1}{2}$ by the argument used in Case 2, and, thus, $\gamma _{(S,x)} = \frac{1}{2} \cdot (\frac{1}{2} + \frac{1}{2}) = \frac{1}{2}$, as required. $\square$

While Theorem 7.2 focuses on full circular split systems, it also suggests two specific phylogenetic diversity indices $\Psi _1$ and $\Psi _2$ for any circular split system that is not full. Consider, as an example, again the split system $\mathscr {S}$ in Fig. 10a. We have $\mathscr {S} \subseteq \mathscr {S}_{\theta }$ for the ordering $\theta = a,b,c,d$. To obtain the complete linear phylogenetic diversity index $\Psi _1$ on $\mathbb {L}(\mathscr {S})$, we restrict the matrix $\Gamma$ obtained for $\mathscr {S}_{\theta }$ by Theorem 7.2 to those rows that correspond to splits in $\mathscr {S}$. The resulting matrix $\Gamma _{\Psi _1}$ is then the matrix in Fig. 10c with $r=0$. To obtain the complete linear phylogenetic diversity index $\Psi _2$ on $\mathbb {L}(\mathscr {S})$ we restrict the matrix $R$ obtained for $\mathscr {S}_{\theta }$ by Theorem 7.2 to those columns that correspond to splits in $\mathscr {S}$. The resulting matrix $R'$ is a matrix that is right inverse to $M(\mathscr {S})$ and we put $\Psi _2 = \Psi _{R'}$. More specifically, the matrix $R'$ equals the matrix in Fig. 10b with $p_1=p_3=\frac{1}{2}$ and $p_2=p_4=p_5=0$ and the matrix $\Gamma _{\Psi _2}$ is the matrix in Fig. 10c with $r=\frac{1}{2}$.

**Fig. 11:**
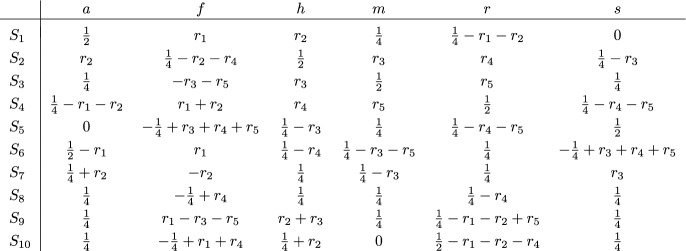
The matrices $\Gamma _{\Psi }$ for the phylogenetic diversity indices $\Psi =\Psi _R$ obtained from the matrices $R$ that are right inverse to the matrix $M(\mathscr {S})$ for the split system $\mathscr {S}$ on $X=\{a,f,h,m,r,s\}$ represented by the network in Fig. 2a. The splits $S_1,\dots ,S_{10}$ are listed in Fig. 12 in the Appendix

**Fig. 12:**
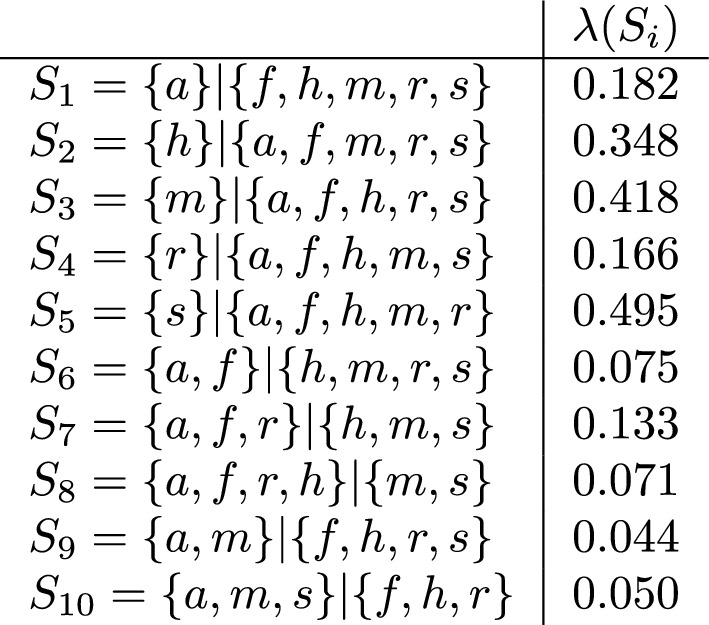
The split system $\mathscr {S}=\{S_1,\dots ,S_{10}\}$ on $X=\{a,f,h,m,r,s\}$ and its weighting $\lambda$ represented by the network in Fig. 2a. The elements in $X$ represent sampling locations of spotted owls in western North America (*a* = Aguascalientes, Mexico; *f* = San Fransisco Peaks, AZ; *h* = Huachuca Mountains, AZ; *m* = Marin County, CA; *r* = Capitol Reef National Park, UT; *s* = San Bernardino Mountains, CA). The weighted split system is computed from the pairwise genetic distances between these populations given in Table S1 of Volkmann et al. (2014) using the implementation of NeighborNet (Bryant and Moulton 2004) in SplitsTree (Huson and Bryant 2005). The same methodology was applied by Volkmann et al. (2014) to all 32 populations in the data set. Here six populations are selected for illustration purposes. The total weight of all splits in $\mathscr {S}$ is $1.980$ (all weights rounded to three decimal places)

**Fig. 13:**
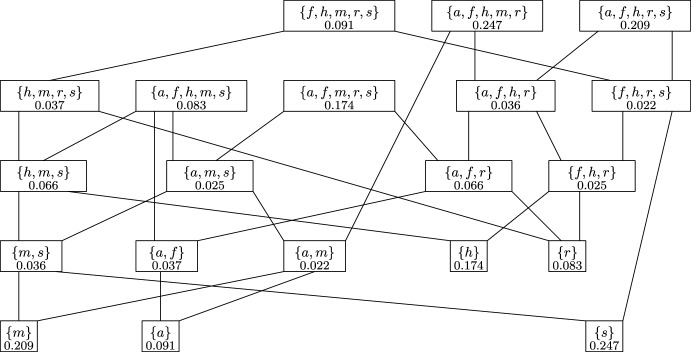
The Hasse diagram for the clusters in the cluster system $\mathscr {C}(\mathscr {S})$ on $X = \{a,f,h,m,r,s\}$ computed from the split system $\mathscr {S}$ on $X$ in Fig. 12. The number below each cluster is the weight of the cluster obtained by the map $\tau$ defined in (17) from the weights of the splits in $\mathscr {S}$

**Fig. 14:**
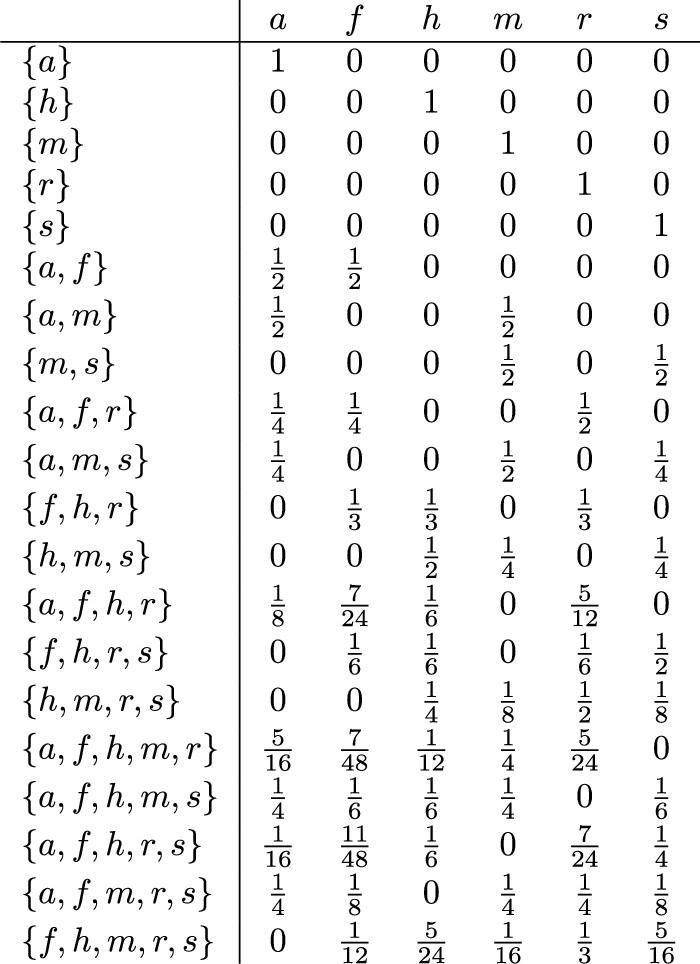
The matrix $\Gamma$ for the equal splits index on $\mathbb {L}(\mathscr {C})$ for the cluster system $\mathscr {C}$ in Fig. 13

**Fig. 15:**
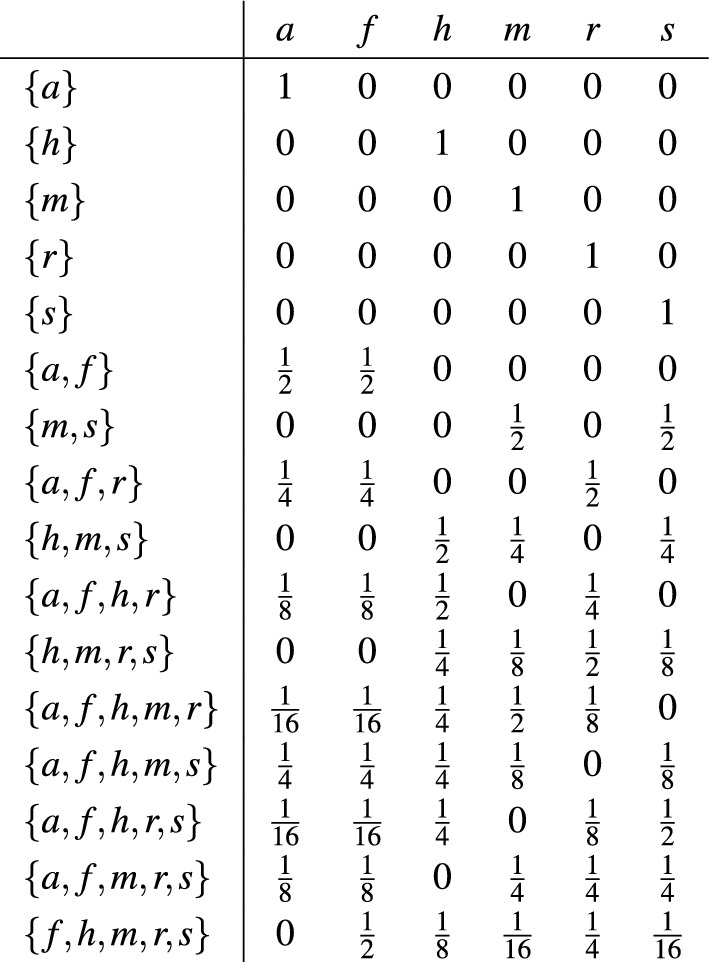
The matrix $\Gamma$ for the equal splits index on $\mathbb {L}(\mathscr {C}_1)$ for the cluster system $\mathscr {C}_1$ obtained from the subset ${{\mathscr {S}}}_1' = \{S_1, S_2, S_3, S_4, S_5, S_6, S_7, S_8\}$ of the split system $\mathscr {S}$ in Fig. 12. Using the map $\tau$ defined in (17) to compute the weights of the clusters in $\mathscr {C}_1$ from the weights of the splits in $\mathscr {S}_1'$, we obtain $\Psi _\tau (s) = 0.450$, $\Psi _\tau (m) = 0.431$, $\Psi _\tau (h) = 0.380$, $\Psi _\tau (r) = 0.267$, $\Psi _\tau (a) = 0.202$, and $\Psi _\tau (f) = 0.156$ (all values rounded to three decimal places)

**Fig. 16:**
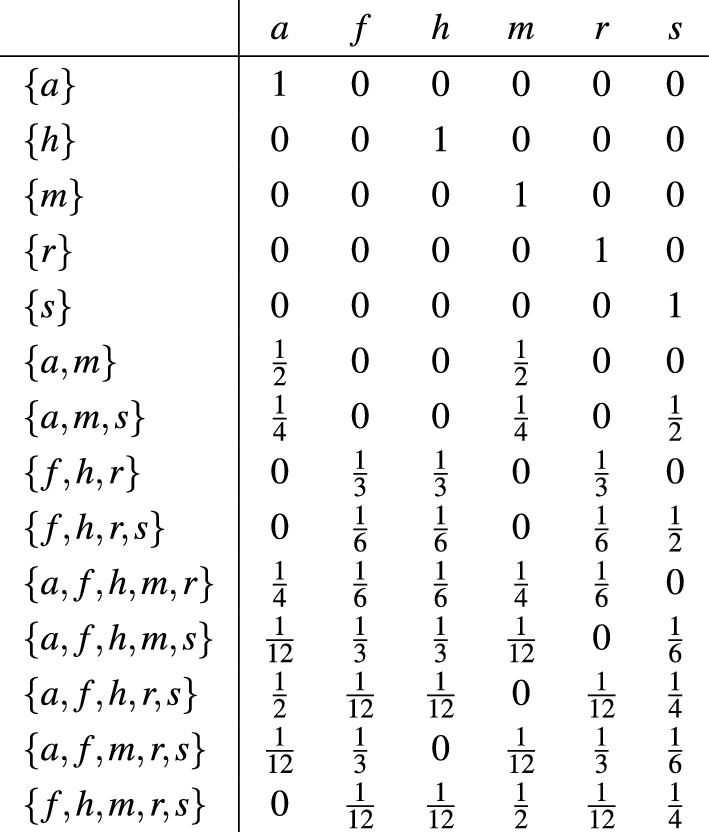
The matrix $\Gamma$ for the equal splits index on $\mathbb {L}(\mathscr {C}_2)$ for the cluster system $\mathscr {C}_2$ obtained from the subset ${{\mathscr {S}}}_2' = \{S_1, S_2, S_3, S_4, S_5,S_9, S_{10}\}$ of the split system $\mathscr {S}$ in Fig. 12. Using the map $\tau$ defined in (17) to compute the weights of the clusters in $\mathscr {C}_2$ from the weights of the splits in $\mathscr {S}_2$, we obtain $\Psi _\tau (s) = 0.388$, $\Psi _\tau (m) = 0.355$, $\Psi _\tau (a) = 0.296$, $\Psi _\tau (h) = 0.280$, $\Psi _\tau (r) = 0.220$, and $\Psi _\tau (f) = 0.164$ (all values rounded to three decimal places)

We conclude this section looking again at the example of owl populations in Fig. 2a. The corresponding split system $\mathscr {S}$ in Fig. 12 in the Appendix is circular with the ordering $\theta =a,f,r,h,s,m$ of the elements in $X$. In Fig. 11 we give the matrices $\Gamma _{\Psi _R}$ obtained from the right inverse matrices $R$ of $M(\mathscr {S})$. They contain five parameters $r_1,\dots ,r_5 \in \mathbb {R}$. Only by putting $r_1=r_4=\frac{1}{4}$ and $r_2=r_3=r_5=0$, however, all entries of $\Gamma _{\Psi _R}$ are non-negative and then $\Gamma _{\Psi _R}$ equals the restriction of the matrix $\Gamma$ obtained for $\mathscr {S}_{\theta }$ by Theorem 7.2 to those rows that correspond to splits in $\mathscr {S}$. Using the weighting $\lambda$ of the splits given in Fig. 12 in the Appendix, we obtain the values of $\Psi _R$ given in Fig. 2b, which yields the same ranking of the six owl populations as $FP_u$ and $SV$.

## Conclusion

In this paper, we have presented a framework for phylogenetic diversity indices defined on linear spaces coming from weighted cluster and split systems. Using some examples of popular tree-based phylogenetic diversity indices from the literature, we have shown that this framework can yield generalizations of these indices for cluster and split systems as well as allowing us to gain a better understanding of their interrelationships.

Note that in our framework presented in Fig. 7, by associating to any split system $\mathscr {S}$ on $X$ the cluster system $\mathscr {C}(\mathscr {S})$ on $X$ and then considering maps $\tau$, we have focused on deriving split-based indices from cluster-based indices. In the affine and projective clustering approach, however, there are also ways to associate to any cluster system $\mathscr {C}$ on $X$ a split system $\mathscr {S}(\mathscr {C})$ on $X$ (see, e.g., Dress 2012, Sec. 9.3). Thus, it could be interesting to investigate how this fact might be used to derive cluster-based indices from split-based indices.

In our results, we have considered cluster and split systems in general, special examples of which include hierarchical cluster systems, compatible split systems (which correspond to rooted and unrooted phylogenetic trees, respectively) and circular split systems. There are, however, various other special classes of cluster and split systems that could be interesting to investigate within our framework. For example, it would also be interesting to consider diversity indices coming from *weak hierarchies*, a special type of cluster system introduced by Bandelt and Dress (1989). The advantage of considering such specialized cluster and split systems is that they can be efficiently computed from biological data, making them potentially more useful for applications.

In the literature, various approaches have been proposed to generalize tree-based phylogenetic diversity indices using phylogenetic networks, a graph-theoretical generalization of phylogenetic trees (Coronado et al. 2018; Volkmann et al. 2014; Wicke and Fischer 2018). Such networks are essentially directed, acyclic, graphs with a single root and whose set of leaves corresponds to some collection of species. The fair proportion index, for example, is generalized in terms of such networks by Coronado et al. (2018). In general, phylogenetic networks give rise to cluster systems (see, e.g., Steel 2016, Sec. 10.3.4) by, for example, taking the set of leaves that lie below a vertex or an edge in the network (just as with rooted phylogenetic trees). Thus, it could be interesting to explore how phylogenetic diversity indices defined in terms of phylogenetic networks, such as, for example, those considered by Wicke and Fischer (2018), fit into our cluster based framework. This could also be interesting to investigate for *split networks* such as the one presented in Fig. 2a, which are a certain type of undirected phylogenetic network (see, e.g., Dress 2012, Sec. 4.4).

With the different ways of defining diversity indices via clusters and splits and translating between the two viewpoints, it could also be interesting to analyze under which circumstances different indices give exactly the same score and thus also identical rankings of the taxa. For example, Wicke and Steel (2020) characterized precisely when the fair proportion index and the equal splits index for rooted binary phylogenetic trees coincide. Thus, it would be interesting to characterize which conditions a weighted cluster system (resp. weighted split system) has to satisfy in order to obtain similar results for pairs of cluster- or split-based indices.

In another direction, it could be interesting to apply our framework to establish properties and generalizations of other tree-based phylogenetic diversity indices that we did not consider in this paper. Indeed, as we have demonstrated, sometimes expressing indices in terms of clusters or splits can lead to more concise proofs for showing that they have certain properties. For example, it would be interesting to consider some of the questions asked in Section 6 of Wicke and Steel (2020) within our new framework.

Finally, concerning the generalization of the Pauplin index presented in Sect. 7, we saw in the examples in Figs. 10 and 11 that even when a circular split system $\mathscr {S}$ is not full there may exist right inverse matrices $R$ for $M(\mathscr {S})$ such that for the complete linear phylogenetic diversity index $\Psi _R$ the matrix $\Gamma _{\Psi _R}$ has non-negative entries. Can we characterize when this is the case and, more specifically, give the number of parameters in the matrix $\Gamma _{\Psi _R}$? As a potential direction for further generalization, one could consider split systems $\mathscr {S}$ for which the matrix $M(\mathscr {S})$ has full rank, which are known as *linearly independent* split systems. There exist such split systems that are not circular (Bryant and Dress 2007). Can Theorem 7.2 be generalized in some way to all maximum sized linearly independent split systems?

## Data Availability

No data was generated.

## Appendix

See Figs. 12, 13, 14, 15 and 16.

## Footnotes

1 https://www.pdtf.org/.

2 https://www.edgeofexistence.org/.

3 In biological applications, weights are usually assumed to be non-negative. Our framework also applies when this assumption is violated.
